# Testing Electrostatic Complementarity in Enzyme Catalysis: Hydrogen Bonding in the Ketosteroid Isomerase Oxyanion Hole

**DOI:** 10.1371/journal.pbio.0040099

**Published:** 2006-03-28

**Authors:** Daniel A Kraut, Paul A Sigala, Brandon Pybus, Corey W Liu, Dagmar Ringe, Gregory A Petsko, Daniel Herschlag

**Affiliations:** **1**Department of Biochemistry, Stanford University, Stanford, California, United States of America; **2**Department of Biochemistry, Brandeis University, Waltham, Massachusetts, United States of America; **3**Stanford Magnetic Resonance Laboratory, Stanford University, Stanford, California, United States of America; University of MichiganUnited States of America

## Abstract

A longstanding proposal in enzymology is that enzymes are electrostatically and geometrically complementary to the transition states of the reactions they catalyze and that this complementarity contributes to catalysis. Experimental evaluation of this contribution, however, has been difficult. We have systematically dissected the potential contribution to catalysis from electrostatic complementarity in ketosteroid isomerase. Phenolates, analogs of the transition state and reaction intermediate, bind and accept two hydrogen bonds in an active site oxyanion hole. The binding of substituted phenolates of constant molecular shape but increasing p *K*_a_ models the charge accumulation in the oxyanion hole during the enzymatic reaction. As charge localization increases, the NMR chemical shifts of protons involved in oxyanion hole hydrogen bonds increase by 0.50–0.76 ppm/p *K*_a_ unit, suggesting a bond shortening of ˜0.02 Å/p *K*_a_ unit. Nevertheless, there is little change in binding affinity across a series of substituted phenolates (ΔΔG = −0.2 kcal/mol/p *K*_a_ unit). The small effect of increased charge localization on affinity occurs despite the shortening of the hydrogen bonds and a large favorable change in binding enthalpy (ΔΔH = −2.0 kcal/mol/p *K*_a_ unit). This shallow dependence of binding affinity suggests that electrostatic complementarity in the oxyanion hole makes at most a modest contribution to catalysis of ˜300-fold. We propose that geometrical complementarity between the oxyanion hole hydrogen-bond donors and the transition state oxyanion provides a significant catalytic contribution, and suggest that KSI, like other enzymes, achieves its catalytic prowess through a combination of modest contributions from several mechanisms rather than from a single dominant contribution.

## Abstract 2

Enzymatic reactions require exquisitely detailed molecular interactions. Here the authors show that geometric complementarity is likely more important than electrostatic charge in contributing to the binding necessary for catalytic reactions.

## Introduction

Chemical transformations catalyzed by enzymes are central to biology, and enzymes have evolved to carry out these transformations with enormous rate enhancements and exquisite specificities. Decades of research have demystified enzymes, elucidating important features of their catalytic power. Numerous structural studies have revealed active sites in crevices or pockets within enzymes. These pockets facilitate specificity by recognizing cognate substrates and by using binding interactions to position substrates for reaction [ 1– 4]. Further, coenzymes, metal ions, and metal clusters that participate in enzymatic reactions are present at active sites, as are side chains that act as general acids and bases to facilitate the proton transfers that are ubiquitous in enzymatic transformations [e.g., 5–8]. Despite these extraordinary advances, a thorough, in-depth, and quantitative understanding of enzymatic catalysis remains a central goal of biochemistry [e.g., 4,9–13 and references therein].

**Figure 1:**
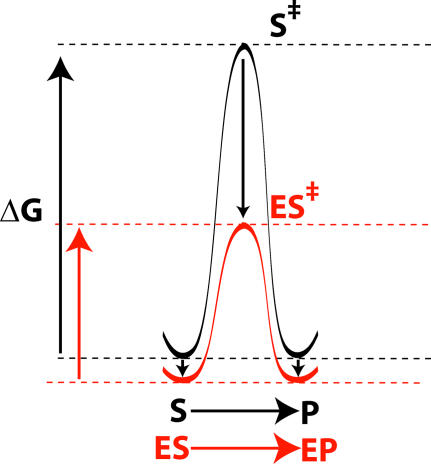
Catalysis Is Preferential Transition State Stabilization

Polanyi, Pauling, and others noted that enzymatic catalysis can be considered as preferential stabilization of a reaction's transition state relative to its ground state [ 14– 19], and indeed this is the definition of catalysis according to transition state theory ( Figure 1) [ 20]. Following from this viewpoint, it was suggested that enzymes provide an environment complementary to electrostatic and geometrical features of the transition state that differ from the ground state and that this complementarity contributes to observed rate enhancements [ 14– 19]. Indeed, this complementarity has been the basis for the development of catalytic antibodies raised against antigens that geometrically and electrostatically resemble transition states [ 21– 23]. The use of this complementarity for catalysis, which has been widely discussed [ 2, 14– 19], is the subject of this paper and is further introduced through the following examples.

**Figure 2:**
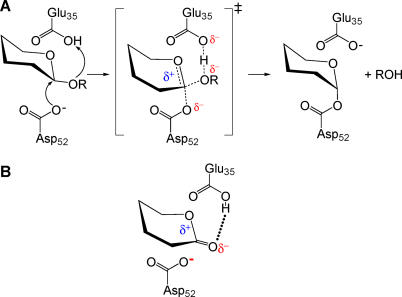
Geometric or Electrostatic Complementarity in the Lysozyme Reaction (A) Simplified mechanism of lysozyme. Asp52 attacks the substrate, and the general acid Glu35 protonates the leaving group oxygen. The reaction proceeds through a loose transition state, in which the bond to the leaving group is nearly broken with only a small amount of bond formation to the incoming aspartate. Instead, positive charge accumulates on the C1 carbon of the sugar and the ring oxygen atom (δ ^+^). This transition state has a half-chair or sofa-like conformation, distinct from the ground state chair conformation [ 31, 36]. (B) Simplified structure of a transition-state analog that binds tightly to lysozyme [ 32]. This analog has the same half-chair conformation as the transition state. It also has a similar charge distribution, with positive charge localized on the carbon and oxygen atoms in the ring and negative charge on the carbonyl oxygen.

Lysozyme, which catalyzes the hydrolysis of bacterial cell wall sugars, is the classic example of an enzyme proposed to accelerate a reaction via geometric complementarity to the transition state [ 24– 31]. Lysozyme's substrate changes shape in the course of reaction, proceeding from a “chair” ground state geometry to a “half-chair” or “sofa” in the transition state ( Figure 2A). Remarkably, a sugar analog with a half-chair geometry bound lysozyme more strongly than the analogously bound substrate by an estimated 6000-fold ( Figure 2B). This 5 kcal/mol preferential binding of a ligand with the transition state's geometry led to the suggestion that geometrical complementarity played a significant role in transition state stabilization [32,33, see also 34,35].

Inspection of features of the lysozyme reaction, however, introduces a distinct possibility, that of enhanced electrostatic complementarity. In the transition state, charge develops on the atoms at the site of reaction—the ring carbon and oxygen atoms develop partial positive charge character, and the leaving group oxygen atom develops partial negative charge character ( Figure 2A) [ 29, 30, 36]. Analogously, the carbonyl group of the transition state analog carries a dipole, with excess positive charge on the ring carbon and oxygen atoms and excess negative charge on the carbonyl oxygen atom, as depicted by the partial charges in Figure 2B [ 33, 35, 37, 38]. Indeed, it has been suggested on the basis of computational results that electrostatic and not geometric complementarity is predominantly responsible for catalysis by lysozyme [ 39] and that electrostatic complementarity is the central hallmark of enzymatic catalysis [ 10, 40]. These conflicting models for lysozyme action reflect the difficulty in disentangling and assessing contributions to catalysis from geometry and electrostatics.

**Figure 3:**
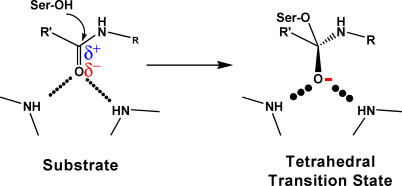
The Serine Protease Reaction Showing Interactions in the Oxyanion Hole

Serine proteases, enzymes that hydrolyze peptide bonds, provide a second classic example in which geometric and electrostatic complementarity are commonly invoked to account for catalysis [ 25, 26, 28, 41– 43]. The peptide substrate to be hydrolyzed undergoes changes in both shape and charge distribution in the course of reaction, proceeding from a planar carbonyl group in the ground state to a tetrahedral transition state with significant buildup of negative charge on the oxygen atom ( Figure 3). This negative charge appears to be stabilized by enzyme hydrogen bonds in what has been termed an “oxyanion hole” [ 44], and mutation of a single oxyanion hole residue leads to a 200-fold rate decrease [45,46, see also 47]. Model building based on early serine protease structures led to the suggestion that the oxyanion hole provided transition state complementarity by donating hydrogen bonds to the tetrahedral transition state's oxyanion, but not to the substrate's carbonyl group, which would not fit geometrically [ 44, 48]. Subsequent structures and vibrational spectroscopy experiments, however, have suggested that substrate carbonyl groups can accept hydrogen bonds in the oxyanion hole [ 49, 50]. Thus, it is possible that the increased charge localization on the oxygen atom leads to a strengthened hydrogen bond in the transition state and that this electrostatic complementarity provides catalysis [ 45, 51, 52].

As the above examples illustrate, the inability to assess and dissect electrostatic and geometric complementarity to the transition state represents an unmet challenge that is fundamental for understanding the basis of enzymatic catalysis. Site-directed mutagenesis, a powerful tool for revealing catalytic residues, can identify potential transition state interactions, such as hydrogen bonds, by the detrimental effects of mutations. A hydrogen bond can be catalytic if it is formed only in the transition state due to geometric changes in the substrate or is formed in both the ground state and transition state but becomes stronger in the transition state due to changes in charge localization. However, removal of the hydrogen-bonding side-chain by site-directed mutagenesis will disrupt catalysis in either case. Similarly, computational techniques that assess the strength of electrostatic interactions will report a stronger interaction in the transition state regardless of whether the effect is due to changes in molecular geometry or charge localization.

An alternative approach would be to attempt to isolate either the geometric or electrostatic features of the reaction, using substrate and transition-state analogs, and determine how interactions with the enzyme depend upon these features. For example, the relative affinities of a series of compounds in which the charge arrangement of the molecule is constant but the geometry is varied between that of the ground state and that of the transition state could report on the role of geometric complementarity to the transition state in a given enzyme. Conversely, the relative affinities of a series of compounds in which the geometry of the molecule is constant but the charge arrangement is varied between that of the ground state and that of the transition state could report on the role of electrostatic complementarity to the transition state. If an enzyme uses geometric complementarity to the transition state for catalysis, it should bind more tightly to analogs with the shape of the transition state (relative to analogs with the same charge distribution but different geometry), and if an enzyme uses electrostatic complementarity to the transition state for catalysis, it should bind more tightly to analogs with similar charge distributions to the transition state (relative to analogs with the same geometry but a different charge distribution).

**Figure 4:**
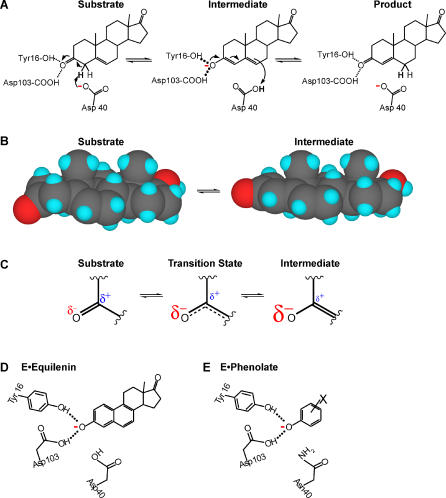
Geometric and Electrostatic Changes in the KSI Reaction (A) Mechanism of KSI catalyzed isomerization of 5-androstene-3,17-dione (substrate) to 4-androstene-3,17-dione (product). In the first step a general base, Asp40, removes a proton from the steroid to form a dienolate intermediate (via a dienolate-like transition state), which receives hydrogen bonds from an oxyanion hole consisting of Tyr16 and protonated Asp103. In the second step of the reaction the steroid is reprotonated at a different position to give the product. (B) Geometric changes accompanying the first half of the KSI reaction. Oxygen is shown in red, carbon in grey, hydrogen in blue. The ring geometry changes in the transition state and in the intermediate, becoming more planar in the intermediate. The sp ^2^-hybridized carbonyl oxygen in the substrate becomes a predominantly sp ^3^-hybridized oxyanion. Structures were generated using CaCHE 4.93 (Fujitsu, Tokyo, Japan) MOPAC PM5 geometry optimization [ 150] and rendered using CS Chem3D Pro 5.0 (CambridgeSoft, Cambridge, Massachusetts, United States). (C) Electrostatic changes at the carbonyl group accompanying the first half of the KSI reaction. The larger “δ−” refers to increased negative charge on the oxygen atom as the reaction proceeds. The dienolate-like transition state is expected to be between the substrate and dienolate intermediate in charge arrangement, but closer to the high-energy intermediate. (D) Schematic depiction of the steroid equilenin bound at the KSI active site. Equilenin geometrically and electrostatically resembles the dienolate reaction intermediate and transition state. (E) Schematic depiction of a single-ringed phenolate bound at the active site of KSI *^D40N^*, the mutant enzyme used for this work. The Asp40Asn mutation mimics the protonated aspartate found in the intermediate and equilenin complexes, see (A) and (D), and leads to tighter binding of phenolate and other intermediate analogs [ 69, 71].

Herein we present a systematic examination that isolates the potential contribution to catalysis from electrostatic complementarity in a particularly tractable system, the bacterial enzyme ketosteroid isomerase (KSI, Figure 4A). We examined the binding to the enzyme of a series of compounds with constant molecular shape but varying charge distribution. The results suggest at most a modest contribution to catalysis from electrostatic complementarity to the transition state via hydrogen bonding in an oxyanion hole. We propose that geometric complementarity may play a significant role in the reaction catalyzed by KSI.

## Results/Discussion

We first describe KSI, an enzyme suggested to provide electrostatic complementarity to the transition state via oxyanion hole hydrogen bonds. We next discuss aspects of hydrogen bonds and their ability to discriminate between the ground state and transition state in an enzyme active site that are critical to the design and interpretation of the experiments herein. We then describe and implement a strategy to experimentally isolate and evaluate the effects of electrostatic complementarity on the reaction catalyzed by KSI.

Two closely related homologs of the bacterial enzyme have previously been characterized, one from Commamonas testosteroni (tKSI) and one from Pseudomonas putida (pKSI); these enzymes are 34% identical and share the same catalytic residues. (For simplicity, pKSI residue numbering is used throughout.) The critical energetic analysis described below was carried out with both enzymes, and identical results were obtained. Further characterization of KSI via X-ray crystallography, NMR, and isothermal titration calorimetry (ITC) was more readily carried out with one or the other enzyme: pKSI for obtaining a high-resolution crystal structure and for calorimetry and tKSI for NMR spectroscopy.

### KSI and Hydrogen Bonding

KSI has an active site oxyanion hole containing two hydrogen-bond donating residues, analogous to serine proteases and many other enzymes ( Figure 4A). As noted in the Introduction, the oxyanion hole can contribute to catalysis via electrostatic and/or geometrical complementarity. The goal of this study was to isolate and systematically evaluate the electrostatic component of catalysis. To our knowledge, such an experimental evaluation has not previously been accomplished at an enzymatic active site.

As for the examples of lysozyme and serine proteases ( Figures 2 and 3), the KSI reaction involves changes in both geometry and charge distribution as the reaction proceeds from its ground state to its intermediate ( Figure 4B and 4C). Site-directed mutagenesis experiments indicate that catalysis by KSI is greatly reduced upon removal of the oxyanion hole hydrogen-bond donors: k _cat_ values are reduced for the Y16F and D103A tKSI mutants by 50,000-fold and 5,000-fold, respectively, representing energetic effects of 6.3 and 5.0 kcal/mol [ 53, 54]. But these results do not allow a parsing of the geometric and electrostatic contributions, as noted in the Introduction. Nor do the rate decreases from site-directed mutagenesis necessarily provide the overall contribution to catalysis from the sum of geometric and electrostatic factors [ 10, 11, 55].

Another approach for determining the electrostatic contribution of hydrogen bonds such as those in the KSI oxyanion hole would use values for hydrogen-bond energetics from simpler nonenzymatic systems. Unfortunately, model studies of hydrogen bonds indicate that their energetic properties are enormously sensitive to the environment [56 and references therein]. For example, the hydrogen bond formed between hydrogen fluoride and the fluoride anion has a free energy change of association (ΔG ^°^) of −0.8 kcal/mol in water, whereas this hydrogen-bonded complex is much stronger in the gas phase, with ΔG ^°^ estimates of −32 to −39 kcal/mol [ 57– 60]. Thus, there are no consensus hydrogen-bond energies that can be applied to enzymatic systems to give quantitative or even qualitative assessments of electrostatic contributions to catalysis.

Several researchers have suggested that the exclusion of water and an organic-solvent or gas-phase–like environment at enzyme active sites could allow the formation of “short, strong” or “low-barrier” hydrogen bonds between enzymes and transition states, leading to substantial preferential transition state stabilization [ 61– 65]. Such hydrogen bonds are normally observed in nonaqueous environments and have unusual physical properties, including short hydrogen-bond lengths, a greatly deshielded proton NMR signal, and partial covalent character [ 66, 67]. Although the energetics of hydrogen-bond formation in the gas phase and organic solvents suggest that strong hydrogen bonds may be possible in an enzyme, an active site provides a unique environment that is distinct from water, organic solvents, and the gas phase. Therefore, it is not possible *a priori* to ascribe specific energetic behavior to hydrogen bonds within an active site.

**Figure 5:**
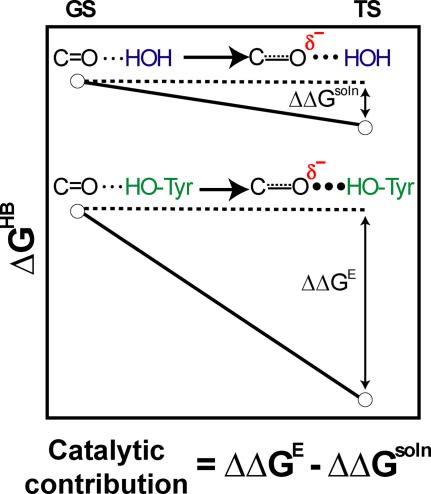
Potential Catalytic Contribution from a Greater Strengthening of Hydrogen Bonds Accompanying Charge Redistribution in an Enzymatic Environment than in Aqueous Solution As charge increases on the carbonyl oxygen going from the ground state to the transition state, hydrogen bonds from either water or an enzyme tyrosine will strengthen. Results from small molecule studies indicate that in a nonaqueous environment such as an enzyme active site, this strengthening (ΔΔG ^E^) can be greater than in aqueous solution (ΔΔG ^soln^), where little strengthening is observed [ 68, 151, 152]. This potential differential strengthening is indicated by the different sizes of hydrogen-bonded dots and the larger change in ΔG between the ground state and the transition state for the enzymatic reaction compared with the solution reaction. Adapted from [ 68].

While the unique enzyme environment precludes simple comparisons, it also provides the potential for another type of catalytic advantage solely from electrostatic interactions, regardless of whether the enzymatic hydrogen bonds may be considered “low barrier” or not. Studies on model hydrogen-bonding systems in nonaqueous environments have revealed a greater sensitivity of hydrogen-bond energetics than in water—i.e., the *change* in hydrogen-bond strength as charge localization changes is greater in nonaqueous environments, as predicted from simple electrostatic considerations [56 and references therein]. An analogous increased sensitivity within active sites could contribute to enzymatic catalysis by providing a larger increase in hydrogen-bond energy in going from the ground state to the transition state at the active site than in water [ 68], as shown schematically in Figure 5. Increased hydrogen-bond sensitivity will only be catalytic if the enzymatic environment, the groups making direct hydrogen bonds and the remainder of the enzyme, provides greater overall electrostatic stabilization of the transition state than is achieved in solution [ 10, 11, 40, 56]. Computational studies have suggested that such preferential electrostatic environments are the norm in enzyme active sites [ 10, 40]. Nevertheless, these computational methods and results require extensive and rigorous experimental validation.

In summary, the contribution of electrostatic complementarity in KSI and other enzymes has yet to be established experimentally and cannot be extracted from model systems. We next describe the experimental approach taken to address this question with KSI.

### Testing Active Site Electrostatic Complementarity with KSI

Pioneering studies by Pollack and coworkers suggested that an evaluation of electrostatic complementarity might be possible using KSI [ 69]. Steroids such as equilenin that resemble the intermediate dienolate in the KSI reaction bind tightly and receive hydrogen bonds in the enzyme's oxyanion hole [ 70– 72] ( Figure 4D). Phenolate anions, which contain a single ring instead of the multiple rings of a steroid ( Figure 4E), also bind to the active site [ 69].

The addition of electron-withdrawing substituents to a phenolate ring changes the p *K*_a_ and charge distribution of the molecule by delocalizing electron density from the phenolate oxygen into the ring and substituents via resonance and inductive effects. This change in charge distribution without concomitant changes in molecular shape can in principle be used as a probe of the oxyanion hole that models the electrostatic changes that occur between the ground state and transition state (Text S1).

**Figure 6:**
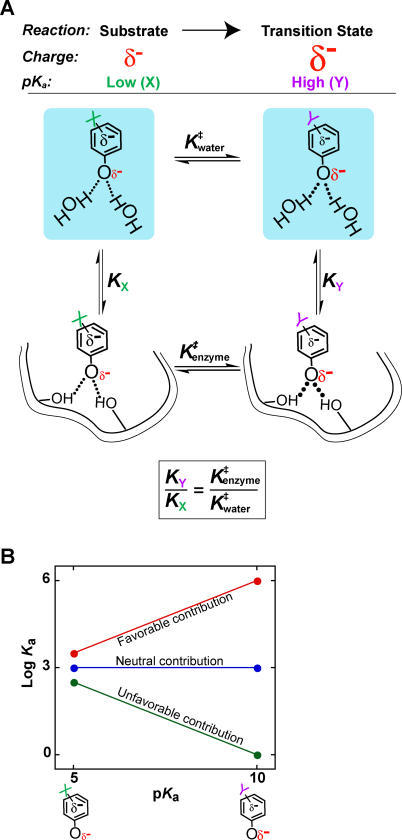
Schematic Depiction of an Experimental Method to Isolate and Probe the Role of Electrostatic Complementarity to the Transition State (A) In the reaction to be probed there is an accumulation of negative charge in the transition state. Two phenolates with different substituents (X and Y) and therefore different p *K*_a_ values and different charge densities on the oxygen atom are used to mimic the increase in charge localization going from the ground state to the transition state. As charge localization on the oxygen increases, hydrogen bonds may strengthen in both water and in the enzyme active site (larger dots for hydrogen bonds formed by phenolate Y). The equilibria and represent the exchange of one phenolate for the other in water and on the enzyme, respectively, mimicking the change in charge localization along the reaction coordinate. The equilibria *K*_X_ and *K*_Y_ are the affinities of the two phenolates, i.e., the equilibrium for their transfer from water to the enzyme. (B) The log of the affinities of the substituted phenolates from (A) are plotted versus p *K*_a_. If the phenolate with substituent Y binds more strongly than that with substituent X (red; *K*_Y_ > *K*_X_), then the enzyme is better than water at stabilizing increased charge localization and there is a favorable contribution to catalysis from electrostatic complementarity. The sign and steepness of the slope, as established using a series of substituted phenolates, can determine the sign and magnitude of the contribution to catalysis.

Figure 6 shows schematically how a series of substituted phenolates can provide information about electrostatic contributions to catalysis. As the reaction catalyzed by KSI proceeds from the substrate to the transition state (and intermediate), negative charge density on the oxyanion increases; this increase is indicated by the increasing size of δ ^−^ in Figure 4C. Analogously, as the p *K*_a_ of a phenolate is raised, there is an increase in charge localization on the oxyanion, as shown for the example of low and high p *K*_a_ phenolates with substituents X and Y, respectively, in Figure 6A. As charge localization increases, hydrogen bonds are expected to strengthen in the oxyanion hole, and this is represented by the larger dots on the transition state side of the 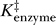 equilibrium. However, hydrogen bonds will also strengthen in solution, and this is shown in the 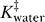 equilibrium. Hydrogen bonds will only be electrostatically catalytic if they lead to greater stabilization of the charge arrangement in the transition state in the oxyanion hole than in water ( 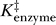
> 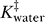). In other words, electrostatic catalysis requires a greater sensitivity to charge localization in the active site than in water. While this sensitivity ( 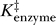/ 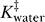) cannot be directly measured, it is equal to *K*_Y_/ *K*_X_, the ratio of the affinities of the two phenolates for the active site; i.e., if the active site is better at stabilizing the localized charge than water is, the higher p *K*_a_ phenolate will bind more strongly ( *K*_Y_ > *K*_X_).

Rather than using only two phenolates, the affinities of a series of compounds with a range of charge localizations has been determined herein. The slope or steepness of the relationship between the log of the affinities and the phenolate p *K*_a_ values reports on the magnitude and sign of any differential sensitivity to charge localization between the enzyme and water and thus on the electrostatic contribution (or barrier) to catalysis. Potential experimental outcomes are shown in Figure 6B in which possible values for binding of the two phenolates from Figure 6A are plotted.

### X-Ray Crystal Structure Shows Phenolate Bound in the Oxyanion Hole

To determine whether phenolates bind in the oxyanion hole in a manner similar to other intermediate analogs, the crystal structure of pKSI *^D40N^* co-crystallized with phenol was solved to a resolution of 1.25 Å (PDB code 2B32). Table 1 shows the crystallographic data collection and refinement statistics. The overall structure is the same as that observed previously for free KSI (PDB code 1OPY) and KSI bound to the intermediate analog equilenin (PDB code 1OGX), with RMS deviations of 0.3 to 0.5 Å for the Cα chains [ 72].

**Table 1:**
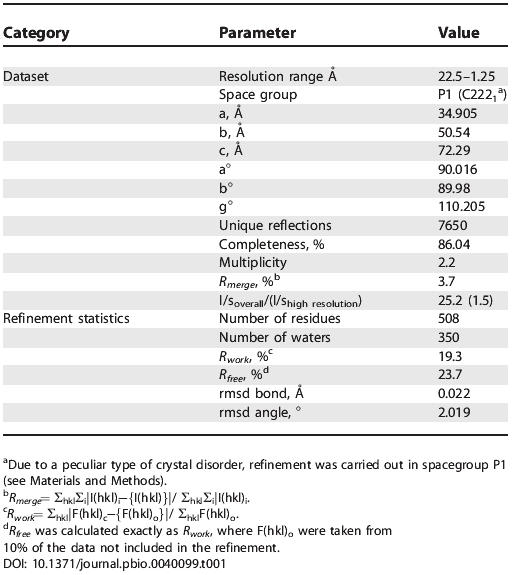
Data and Refinement Statistics for pKSI *^D40N^*•Phenolate

**Figure 7:**
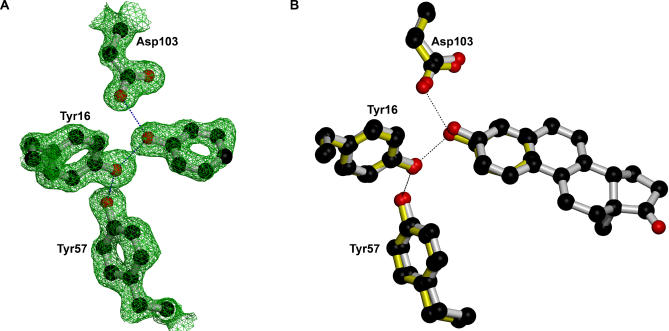
Crystal Structure of Phenolate Bound at the Active Site of pKSI *^D40N^* (A) Electron density map (1.5 σ) shows that phenolate is bound at the oxyanion hole, receiving short hydrogen bonds from Tyr16 and Asp103. (B) Overlay of the phenolate•KSI structure (yellow) and the intermediate analog equilenin•KSI structure (grey; PDB code 1OGX [ 72]) at the active site.

Results with several spectroscopic techniques confirmed that the anionic phenolate form of the ligand, with a negatively charged oxygen, was bound rather than the unionized phenol form (Text S2). The bound phenolate ligand is nearly superimposable with the oxyanion-containing ring of the intermediate analog equilenin ( Figure 7) [ 72]. The phenolate oxygen atom is positioned to receive short hydrogen bonds from Tyr16 and Asp103 ( Table 2). As described in Materials and Methods, the KSI•phenolate co-crystals were solved in the P1 space group due to a peculiar type of crystal disorder, which did not allow for adequate refinement in the typically reported space group C222 _1_. However, this approach allowed four independently refined determinations of the structure and the distances in the oxyanion hole. There is excellent agreement between the complexes (RMSD values of 0.067–0.071 Å), and the corresponding oxyanion hole hydrogen-bond distances are within 0.06 Å of one another. The average distances of 2.49 and 2.61 Å from the phenolate oxygen to the Tyr16 and Asp103 oxygen atoms, respectively, are similar to the corresponding distances reported to the oxygen atom of bound equilenin of 2.55 and 2.56 Å [ 72]. These distances are shorter than the vast majority of hydrogen bonds in enzyme and small molecule complexes and approach the shortest reported O–O hydrogen-bond distances of ˜2.4 Å [ 66, 73].

**Table 2:**
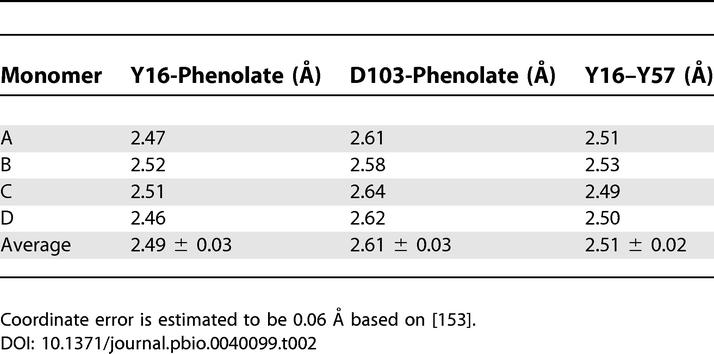
Oxyanion Hole O–O Hydrogen-Bond Lengths

### Hydrogen Bonds Shorten with Increasing Charge Localization

As noted above, shortened hydrogen bonds in the transition state have been suggested to result in favorable hydrogen-bond energies in enzyme active sites during catalysis [ 62, 63]. Do hydrogen bonds in an enzyme active site shorten as charge localization increases? The binding of phenolates provided an opportunity to test this physical behavior.

Unusual physical properties are observed for short O–O hydrogen bonds, including a heightened sensitivity in the position of the bridging proton to changes in O–O distance and far downfield ^1^H NMR chemical shifts [ 66, 73, 74]. For such hydrogen bonds, a strong correlation is observed between the chemical shift of the bridging proton and hydrogen-bond length, in terms of both oxygen–oxygen and oxygen–hydrogen distances [ 75– 77]. Phenolates and other intermediate analogs, when bound to KSI, give rise to far downfield chemical shifts, consistent with formation of short hydrogen bonds [ 69, 78]. Changes in lengths of these hydrogen bonds within the active site can therefore be assessed by changes in their NMR chemical shifts. We first describe the assignment of the observed chemical shifts to specific protons and then present the trend in chemical shift and estimated change in hydrogen-bond length as a function of the p *K*_a_ of the bound phenolate ion.

**Figure 8:**
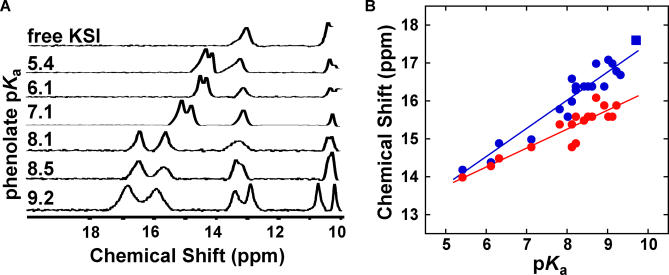
^1^H NMR Downfield Chemical Shifts for Substituted Phenolates Bound to tKSI *^D40N^* (A) Representative spectra, with phenol p *K*_a_ shown on the left. From top to bottom: free enzyme, 3,4-dinitrophenol, 3-fluoro-4-nitrophenol, 4-nitrophenol, 3-fluoro-5-trifluoromethylphenol, 3,4-dichlorophenol, and 3-iodophenol. (B) Correlation between increasing phenolate p *K*_a_ and increasing chemical shift of observed downfield peaks. Circles are the two downfield peaks observed for phenolate binding. A linear fit gives slopes of 0.76 ± 0.06 and 0.50 ± 0.06 ppm/p *K*_a_ unit for the most downfield (blue) and the second-most downfield (red) peak, respectively. The square is the main downfield peak observed with the intermediate analog equilenin (p *K*_a_ = 9.7), for comparison.

The one-dimensional ^1^H NMR spectrum of unliganded tKSI *^D40N^* displays a single peak at ˜13 ppm in the far downfield (>11 ppm) region of the spectrum. Upon binding of substituted phenolates, two new downfield peaks appear at >14 ppm ( Figure 8A). The appearance of these peaks upon phenolate binding and their sensitivity to phenolate p *K*_a_ is readily accounted for by the two hydrogen bonds to bound phenolate from Tyr16 and (neutral) Asp103 that constitute the enzyme's oxyanion hole ( Figures 4E and 7A). This assignment was tested via 2D ^1^H NOESY NMR experiments that identify hydrogen atoms that are close in space, mutations of the oxyanion hole residues, and comparison to model compound chemical shifts (Text S3). The results strongly support assignment of the two downfield peaks to the Tyr16-phenolate and Asp103-phenolate hydrogen bonds.

As the p *K*_a_ of the phenolate increased from 5.4 to 9.3, increasing negative charge density at the phenolate oxygen, the hydrogen-bonded protons became progressively deshielded and their chemical shifts increased from 14.2 to 17.0 ppm ( Figure 8B, Table S1). The 17.6-ppm chemical shift of the principal downfield peak observed upon binding of the steroidal intermediate analog equilenin (p *K*_a_ 9.7, spectrum not shown) is consistent with the hydrogen-bond behavior observed for the phenolates, as shown by the blue square in Figure 8B. The slopes for the change in chemical shift with increasing p *K*_a_ are 0.76 ± 0.06 and 0.50 ± 0.06 ppm/p *K*_a_ unit for the two hydrogen-bond peaks.

Extensive small molecule studies involving O-H–O hydrogen bonds have demonstrated that as the O–O distance decreases from ˜2.70 to 2.45 Å, the chemical shift of the bridging proton increases from 12 to 20 ppm [ 74, 77, 79, 80]. Thus, the observed variation in chemical shift of the KSI-phenolate hydrogen bonds strongly suggests that these hydrogen bonds shorten progressively with increasing phenolate p *K*_a_ [ 76, 81]. The chemical shift/bond length correlations cited above, when applied to KSI, give estimates for hydrogen-bond shortening of ˜0.02 Å per p *K*_a_ unit (Text S4). The O–O distances estimated for phenolate (p *K*_a_ = 10.0) from Figure 8B and these correlations are 2.50 and 2.53 Å, similar to the average values observed in the X-ray structure of 2.49 and 2.61 Å ( Figure 7A). Nevertheless, the predicted change in O–O distance over the p *K*_a_ range of the substituted phenolates assayed in Figure 8 is less than 0.1 Å, a difference difficult to reliably measure by X-ray crystallography.

The 0.02 Å/p *K*_a_ unit change in hydrogen-bond length observed for substituted phenolates is significant on the distance scale of a hydrogen bond. Indeed, considering the **˜**16 unit change in p *K*_a_ of the KSI substrate oxygen between the ground state and the intermediate [ 82], oxyanion hole hydrogen bonds donated to the substrate could shorten by as much as 0.3 Å in the course of the KSI reaction as a result of the electrostatic changes at the oxygen in the oxyanion hole.

Ab initio calculations suggest that shortening a hydrogen bond by 0.02 Å increases the hydrogen-bond strength by 1.7 kcal/mol in the gas phase [ 83, 84]. Nevertheless, the energetic consequences of changing hydrogen-bond character within the idiosyncratic active site environment cannot be predicted. We therefore turned to direct measurement of the change in binding affinity as a function of phenolate p *K*_a_.

### Binding Affinity Is Weakly Sensitive to Increasing Charge Localization

The ability of KSI to bind a series of substituted phenolates in its active site introduced the possibility of isolating and assessing the effect of charge localization within the oxyanion hole on binding affinity ( Figure 6). However, substituents that alter the phenolate p *K*_a_ can also introduce steric or hydrophobic interactions with the enzyme, and these factors can obscure effects from electrostatics alone. Previous results from Pollack and coworkers suggested that hydrophobic effects were indeed convoluted with the electrostatic effects [ 69]. Our initial work demonstrated that a series of phenolates with substituents chosen considering only p *K*_a_ variation did not readily allow extraction of the electrostatic dependence and, further, that association constants determined by the existing assays were limited in accuracy and precision ( Materials and Methods; Text S5). We therefore developed a new binding assay and applied this assay in search of a series of phenolates that would report cleanly on electrostatic effects without substantial interference from steric and hydrophobic factors.

**Figure 9:**
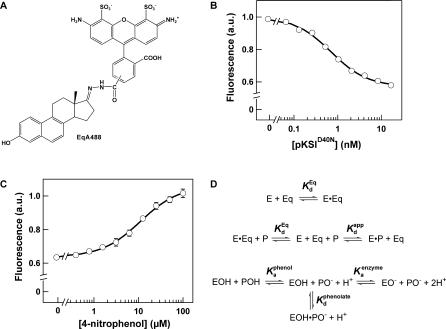
Binding Assay for Phenolates via Competition with a Fluorescently Labeled Equilenin (EqA488–1) (A) Structure of EqA488. Synthesis resulted in two isomers, and the isomer referred to as EqA488-1 was used in further experiments, as described in Materials and Methods. (B) Addition of pKSI *^D40N^* (E) to 0.1 nM EqA488–1 (Eq) (pH 6.9) leads to quenching of fluorescence at 515 nm (excitation at 480 nm). Each point is the average of two replicates (with errors smaller than the points). Data were fit to Equation 1 and gave = 0.7 ± 0.1 nM for this determination. Additional replicates gave = 1.0 ± 0.3 nM. The accuracy of this determination does not impact the comparison of the affinities of the substituted phenolates relative to one another. (C) Addition of 4-nitrophenol (P) to a solution of 0.1 nM EqA488 and 5 nM pKSI *^D40N^* pH 6.9 leads to recovery of fluorescence. Data were fit to Equation 2 and gave = 11.0 ± 0.9 μM. This observed affinity was first converted to an apparent affinity (= 1.8 ± 0.2 μM; Equation 3) of the phenol for the enzyme at (pH 6.9). This apparent affinity was then converted into the pH-independent affinity ( = 26 ± 2 nM; Equation 4) of the phenolate form of the ligand (PO ^−^) for the protonated form of the enzyme (EOH) using the known phenol and enzyme ionization constants ( = 7.1 and = 5.5, respectively; see Materials and Methods). (D) Binding schemes from which Equations 1–4 were derived.

Figure 9 shows the competitive binding assay developed and employed herein. A fluorescent analog of equilenin, referred to as EqA488–1, was synthesized by covalently attaching a dye at the C17 position ( Figure 9A), which faces out of the active site in the KSI•equilenin structure [ 72]. The fluorescence of EqA488–1 was substantially quenched upon binding to KSI ( Figure 9B), and a dissociation constant of 1.0 ± 0.3 nM for pKSI *^D40N^* (pH 6.9) and 1.2 ± 0.1 nM (pH 8.0) was obtained, in agreement with the less precise 0.1–1 nM affinity of equilenin estimated by previous assays [ 71]. Upon addition of phenolates or other active site ligands, EqA488–1 fluorescence increased, indicating displacement from the active site ( Figure 9C). With the EqA488–1 affinity known, the phenolate dissociation constant could then be determined from the concentration dependence for EqA488–1 displacement and known and measured phenol and enzyme p *K*_a_ values using the binding scheme of Figure 9D, as described in Materials and Methods.

Investigation of phenolates with nearly identical p *K*_a_ values but varying alkyl substituents in the *meta*- and *para*-positions revealed a large hydrophobic contribution to binding (Text S5, Figure S1A and S1B). In addition, halogen substituents at the *meta*- and *para*-positions had effects on binding that were strongly correlated with size, with larger substituents at either position leading to increased affinity ( Figure S1C). However, this size effect appeared to be negligible when fluorine, the smallest halogen substituent, was compared with hydrogen on the parent unsubstituted phenolate ( Figure S1D). Fortunately, fluorine substitution at the *para*-position does not perturb the phenolate p *K*_a_. Thus, the identical affinity of phenolate and *para*-F phenolate strongly suggested that a F-substituent at the *para*-position has no significant steric or hydrophobic effect, relative to –H. The similar effects of alkyl or halogen substituents at the *meta*- and *para*-positions further suggested that these potentially complicating effects are also absent for –F substitutions at the *meta*-position. We therefore investigated the binding of a series of phenolates with varying degrees of –F substitution at the *para-* and *meta-*positions.

**Figure 10:**
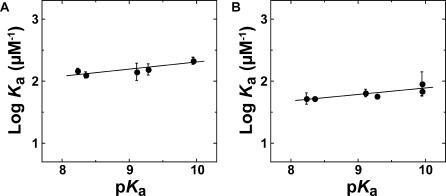
Dependence of the Affinity of a Series of *meta-* and *para*-Substituted Fluorophenolates for KSI *^D40N^* on p *K*_a_ Data for pKSI *^D40N^* is shown in (A) and data for tKSI *^D40N^* is shown in (B), with slopes of 0.11 ± 0.03 and 0.10 ± 0.03, respectively.

Figure 10 shows the dependence of the association constant (log *K*_a_) on the p *K*_a_ of the substituted phenolate in this series for KSI *^D40N^* from both P. putida and C. testosteroni. In both cases the dependence is very shallow, with a slope of only ˜0.1. Substantial data support the conclusion of a shallow dependence, as follows: (i) The same slope is observed in the dependence for enzymes from two different sources, 0.11 ± 0.03 for pKSI *^D40N^* and 0.10 ± 0.03 for tKSI *^D40N^* ( Figure 10A and 10B, Table S2); (ii) Determination of affinity constants by calorimetry (see Materials and Methods and below) for pKSI *^D40N^* gave the same affinities within error as determined by the fluorescence competition assay and a slope of 0.02 ± 0.04 ( Figure S2); (iii) An additional series of phenolates with –F substitutions at the *meta-* and *para-* positions but also all containing a single *ortho*-F substituent gave similar shallow dependences. Although the *ortho*-F substitution weakens binding slightly and shifts the p *K*_a_ down by approximately one p *K*_a_ unit, the series of phenolates all containing this substitution give a linear dependence in plots like that of Figure 10 with slopes of −0.11 ± 0.09 and 0.08 ± 0.06 for pKSI *^D40N^* and tKSI *^D40N^*, respectively ( Figure S3).

The highest p *K*_a_ phenolate, with the highest degree of charge localization in the oxyanion hole and shortest hydrogen bonds, binds only 1.5-fold stronger than that with the lowest p *K*_a_ ( Figure 10). The shallow slope of the correlation between the log of the affinity constant and the p *K*_a_ of the phenolate indicates that the oxyanion hole of KSI is only slightly better able to stabilize increased charge localization than water ( Figure 6). There is a p *K*_a_ difference of 16 between the ground state and the intermediate in the KSI reaction [ 82]. Given the limiting values of the observed shallow sensitivity to charge localization, rate effects from electrostatic complementarity in the oxyanion hole can be estimated that range from a 300-fold (3.4 kcal/mol) enhancement above the aqueous uncatalyzed reaction rate to a 6-fold (1.0 kcal/mol) inhibition (Text S6). The estimated catalytic contribution is small relative to the overall preferential transition state stabilization 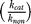 of ˜16 kcal/mol [ 85]. It is also much less than the ˜11 kcal/mol stabilization observed relative to the acetate catalyzed reaction, where acetate provides the same general acid/base functionality as the enzyme active site [ 71, 86].

It remains possible that the electrostatic contribution is larger in the KSI reaction with the full steroid bound instead of with phenolates that contain only one six-membered ring ( Figure 4). However, the ^1^H chemical shift of the equilenin transition state analog falls on the same correlation line as the phenolates, suggesting that the phenolates have the same hydrogen-bond lengths as steroids with corresponding p *K*_a_ values ( Figure 8B). Further, the nearly identical positioning of equilenin and phenolate within the active site ( Figure 7B) suggests that the additional rings present in steroid ligands do not substantially alter binding interactions within the oxyanion hole and that hydrogen bonding interactions for phenolates and the larger steroids are similar.

### Probing the Energetics Underlying the Change in Hydrogen-Bond Character with Increasing Charge Localization

Hydrogen bonds in the active site appeared to shorten as charge localization increased ( Figure 8). Such bond shortening is typically assumed to be accompanied by an increase in bond strength, yet the bond shortening in KSI resulted in only modestly stronger binding ( Figure 10). Most simplistically, bond shortening is expected to be expressed as an enthalpic contribution, which is related to the potential energy of the system, whereas the overall favorability of a process such as phenolate binding is expressed by its free energy change, which contains contributions from enthalpic and entropic changes (ΔG = ΔH −TΔS). We therefore sought to determine the underlying enthalpic and entropic components of phenolate binding that led to a modest increase in affinity with increasing charge localization.

In practice, observed values of ΔH for reactions in solution are complex, including competing contributions from changes in the molecules of interest as well as the surrounding solvent. These changes include formation of hydrogen bonds and other enzyme/ligand contacts, desolvation of the active site, desolvation of the ligand, and rearrangements of the enzyme and ligand upon binding to one another. Thus, measurements of ΔH° alone provide limited physical and molecular information.

Because we had a series of related ligands, the *meta*- and *para*-F-substituted phenolates, a different, more controlled approach was possible. Comparing values of ΔH° eliminates contributions to the binding enthalpy from desolvation of the enzyme active site, interactions with the phenolate ring, and enzyme or ligand rearrangements that occur except for those that change in response to changing phenolate charge density. We therefore determined the *change* in ΔH, ΔΔH, over the series of substituted phenolates to isolate the component of the enthalpy change arising from the change in charge density in the oxyanion hole, relative to the corresponding change in water.

Isothermal titration calorimetry (ITC) was used to determine the relative enthalpies of binding of the series of F-substituted phenolates to the pKSI *^D40N^* enzyme. As noted above, the affinities measured by ITC were very similar to those determined by fluorescence ( Figure S2). Further, replacement of hydrogen with fluorine at the *para-*position of phenolate, which has no effect on the p *K*_a_, gave an identical value of ΔH _bind_ ( Table S3). This result, combined with the small steric and hydrophobic effects of *meta-* and *para-*fluorine substitutions on ΔG ( Figure S1), suggests that any nonelectrostatic contribution to the relative enthalpies of binding of the F-substituted phenolates is negligible.

**Figure 11:**
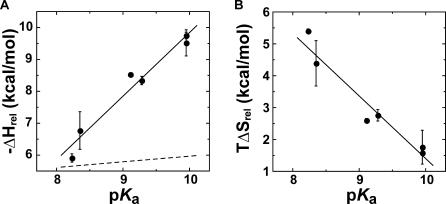
Dependence of Changes in Enthalpy and Entropy of Binding of a Series of *meta-* and *para*-Substituted Fluorophenolates to pKSI *^D40N^* on p *K*_a_ (A) The relative value of ΔH _binding_ as a function of p *K*_a_ (uncorrected for the enzyme ionization enthalpy, which is constant across the series of phenolates) has a slope (solid line) of −2.0 ± 0.2 kcal/mol/p *K*_a_ unit. The dotted line is the relative dependence of −ΔG on p *K*_a_ from Figure 6 for comparison (−0.2 kcal/mol/p *K*_a_ unit). (B) The relative TΔS _binding_ as a function of p *K*_a_ has a slope of 2.0 ± 0.3 kcal/mol.

As was the case for the measurements of overall binding ( Figure 10), the enthalpic data fit well to a linear dependence on p *K*_a_ ( Figure 11A, Table S3). In contrast to the overall binding data, however, the slope of the dependence of ΔH _binding_ on p *K*_a_ was very steep, −2.0 ± 0.2 kcal/mol per p *K*_a_ unit, indicating that binding enthalpy becomes increasingly favorable with increasing charge localization on the phenolate oxygen. As noted above for the correlation with overall binding affinity, a series of *meta*- and *para*-substituted fluorophenolates each containing a single *ortho*-F substituent can also be investigated as a homologous series. The dependence of ΔH on p *K*_a_ for these compounds was the same, within error, as that for the above series (slopes of −2.0 ± 0.4 and −2.0 ± 0.2 with and without the *ortho*-F substitution, respectively; Figure S3C), strongly supporting the conclusion of a steeper dependence of ΔH on phenolate p *K*_a_. The shallow dependence of binding affinity (ΔΔG = −0.2 kcal/mol) in spite of the steeply favorable dependence of binding enthalpy (ΔΔH) implies an offsetting entropic penalty that increases in magnitude with increasing charge localization [Δ(TΔS); Figure 11B].

The calorimetric data indicate that the enthalpic component of binding becomes progressively more favorable as the charge density at the phenolic oxygen increases and as the hydrogen bond shortens. This trend is consistent with the simplest expectations for shortened hydrogen bonds [ 66], although differential rearrangements of the enzyme upon binding also likely contribute to the observed values of ΔΔH and Δ(TΔS), as described below. Further, the large slopes observed for ΔΔH and Δ(TΔS) indicate that there are substantial differences in the energetic response to increased charge localization between the enzyme active site and aqueous solution.

### Conclusions and Implications

Results from site-directed mutagenesis experiments and computation have led to the suggestion that KSI oxyanion hole hydrogen bonds strengthen in the transition state as negative charge is localized on the carbonyl oxygen, thereby making a large electrostatic contribution to catalysis [ 53, 54, 87– 90]. This catalytic strategy, proposed for KSI and many other enzymes, can be described as operating via electrostatic complementarity to the transition state. Nevertheless, the substrate for the KSI reaction, as for other enzymatic reactions, changes in both charge distribution and geometry as the reaction proceeds from its ground state to its transition state ( Figure 4), preventing parsing of energetic contributions into components from electrostatic and geometrical complementarity. Furthermore, hydrogen bonds are highly sensitive to their surrounding environment [56 and references therein] so that the energetics of hydrogen bonds in the KSI oxyanion hole will depend on the enzymatic environment.

#### Electrostatic and geometric complementarity in catalysis by KSI

The ability of KSI to bind phenolate ions in a mode analogous to its reaction intermediate and transition states provided an exceptional opportunity to isolate the electrostatic behavior of the KSI oxyanion hole and thereby evaluate the energetic contribution from electrostatic transition state complementarity. The shallow dependence of the affinity of substituted phenolates for KSI on p *K*_a_, observed with enzymes from two different sources and by two independent binding assays, suggests that electrostatic complementarity in the oxyanion hole provides at most a modest catalytic contribution of ˜300-fold.

**Figure 12:**
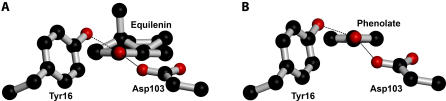
Structure of Equilenin (1OGX) and Phenolate Bound to pKSI*^D40N^*, Showing the Hydrogen Bonds from Tyr16 and Asp103 That Come from Above and Below the Plane of the Steroid (A) For equilenin (PDB Code 1OGX [72]), average angles out of the plane of the steroid ring are 28° and 46°. (B) For phenolate, the average angles are 15° and 47°.

Given the apparently modest contribution from electrostatic transition state complementarity and the possibility for catalysis from geometrical complementarity, it would be desirable to vary geometrical features of reaction coordinate analogs independent of changes in electrostatic properties. Such an experiment would provide the converse of the experiment herein that varied electrostatics while keeping geometry fixed. However, to our knowledge this formidable challenge remains to be met for an enzymatic system. Nevertheless, structural features of the bound oxyanion species observed in this and previous work ( Figure 12, [ 72]) suggest that geometrical changes in going from the ground state to the transition state may lead to preferential transition state interactions [see also 3,15,29,44,91–97].

Hydrogen bonds to sp ^2^ and sp ^3^ hybridized oxygen acceptors have different geometric preferences. Oxygen atoms with sp ^2^ hybridization, such as the carbonyl oxygen of the KSI substrate, preferentially accept hydrogen bonds in the plane of the carbonyl group and steroid whereas sp ^3^ hybridized acceptors, such as the dienolate oxyanion of the intermediate, preferentially accept hydrogen bonds out of this plane [ 98– 103]. In the crystal structures of the intermediate analog equilenin and phenolates bound to pKSI *^D40N^*, the oxyanion accepts hydrogen bonds from above and below the plane of the steroid and phenolate rings ( Figure 12 [ 72] and unpublished data). We propose that active site binding interactions position the incipient oxyanion suboptimally to accept hydrogen bonds in the sp ^2^ ground state and optimally in the sp ^3^ transition state, thereby providing preferential transition state stabilization and catalysis. Crude estimates from empirically derived hydrogen-bonding potentials suggest that contributions on the order of 10 ^2^-fold to 10 ^3^-fold may be possible from such a mechanism [101,103, unpublished data].

It has also been suggested for serine proteases and other enzymes that binding interactions away from the site of chemical transformation position the substrate such that active site hydrogen bonds are prevented from achieving their optimal length in the ground state. As the reaction proceeds and bonds at the reactive center lengthen (or shorten), hydrogen-bond lengths may then become optimal in the transition state, providing additional geometrical catalysis in the active sites of KSI and other enzymes [ 44, 48, 93– 96].

In summary, electrostatic and geometrical complementarity may each play modest roles in catalysis by KSI, and there may be additional contributions from better accommodation of the steroid ring geometry in the transition state than in the ground state. Certainly general base catalysis contributes to the observed rate enhancement of ˜10 ^11^-fold relative to the uncatalyzed reaction in water 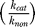 [ 85], with the enzyme providing the general base and positioning it for proton abstraction. Thus, an overall picture emerges, consistent with suggestions for other enzymes [e.g., 31,104–106], in which KSI achieves its substantial rate enhancement through a combination of several modest catalytic contributions.

#### The KSI active site environment

Hydrogen-bond energetics are extremely sensitive to the environment in which they are formed. Thus, the energetic and physical behavior observed for hydrogen bonds in the oxyanion hole of KSI can be used to evaluate the active site environment. Three broad classes of active site environments can be considered, and each has been proposed in a variety of forms in the literature.

Enzyme active sites have been likened to an apolar organic solvent or the gas phase, environments in which electrostatic interactions such as hydrogen bonds to charged or polar transition states are stronger than in water in the sense that complex formation driven by these interactions is more favorable in nonaqueous media than in water [ 56, 61, 63– 65, 107, 108]. However, there is a large desolvation penalty upon transferring charged and polar groups from water to a nonpolar environment [ 109– 112]. Indeed, calculations suggest that such a desolvation penalty would overwhelm favorable hydrogen-bonding interactions such as those in an oxyanion hole [ 40, 113– 115], leading to a net electrostatic inhibition of catalysis. These results are supported by the common expectation that anionic hydrogen-bonded inter- or intra-molecular complexes will not preferentially partition from water into a nonpolar solvent. It remains possible, as described previously, that hydrogen bonds in an organic solvent-like active site become partially covalent and that this covalency gives additional transition state stabilization, leading to a positive contribution to catalysis [ 61, 63– 65].

A second model posits the opposite of a hydrophobic active site. That is, the numerous dipoles from each amide bond and from many side chains may allow active sites to solvate the charge arrangement present in the transition state better than water. Pre-orientation of enzyme dipoles to preferentially stabilize the transition state charge arrangement would provide a catalytic advantage, relative to water, as water molecules must rearrange in order to stabilize charge rearrangements [ 10, 16, 19, 40, 116– 119]. However, there are many more water molecules in a volume of bulk solution to interact with a ligand than there are dipoles in the corresponding volume of an enzyme interior, and it remains unclear how precisely pre-oriented these dipoles are.

A third class of models recognizes that active sites, although often enclosed, are not sequestered far from bulk solvent and that the properties of a nearby high dielectric environment will greatly influence the bulk electrostatic behavior of neighboring low dielectric environments [ 117, 120– 124]. According to these models the exchange of hydrogen bonds between water and the transition state for hydrogen bonds between the enzyme and the transition state would be close to isoenergetic, leading to little if any catalytic advantage from hydrogen bonding [ 25, 125– 127].

While these limiting classes of models are useful conceptually, it is important to recognize that the idiosyncratic environment of an active site is enormously complex and cannot be summarized by a single parameter such as an effective dielectric constant [ 122, 128]. Aspects from each of the above models, desolvation from and loss of hydrogen bonds to bulk water, dipoles within the enzyme and their orientation and mobility, and contributions from the solvent surrounding the enzyme's active site will contribute to the overall observed energetic properties. For example, some rate advantage for KSI catalysis is expected from pre-orientation of the hydrogen-bond donating groups and from the fact that the oxyanion hole hydrogen-bond donors, TyrOH and AspCOOH, are better hydrogen-bond donors than water [ 56]. These factors presumably contribute to the slope observed for the binding of fluorophenolates to KSI, but whether these factors aid catalysis in conjunction with other favorable active site features or offset active site features that would otherwise be inhibitory could not have been determined in the absence of a systematic experimental test as carried out herein.

**Figure 13:**
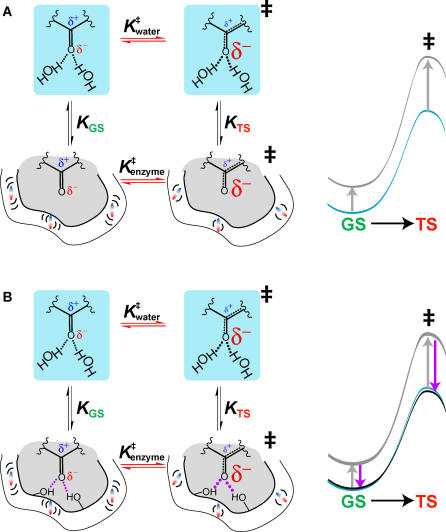
Model for the KSI Active Site Environment and Energetic Consequences Charge accumulates on the oxyanion in the transition state. Transfer from water to a mock active site (or mutant) lacking hydrogen bonds (A) results in a desolvation penalty that is larger in the transition state than in solution as the KSI active site provides only a limited number of dipoles (colored bars) partially pre-oriented to stabilize the transition state charge distribution. Hydrogen bonds, when added back to the active site (B), shorten and strengthen (larger dots and purple arrow) in the transition state on the enzyme, as suggested by the large value of −ΔΔH. Enzyme dipoles rearrange and become more conformationally restricted in the transition state, shown in both (A) and (B), as suggested by the large value of Δ(TΔS). The energetic sum of these interactions results in a free energy reaction profile similar to that in solution.

Figure 13 depicts a simplified active site model to explain the hydrogen-bond behavior in the KSI active site relative to aqueous solution; i.e *.*, the small overall energetic effect (ΔΔG near zero; Figure 10), the steep increase in −ΔH, the steep decrease in TΔS ( Figure 11), and the decrease in oxyanion hole hydrogen-bond length ( Figure 8) with increasing phenolate p *K*_a_. According to this model, the KSI active site provides only a limited number of dipoles (colored bars) that are somewhat pre-oriented to stabilize the transition state charge distribution. Therefore the desolvation penalty incurred upon transferring charge out of water is not replaced by more favorable interactions with multiple preorganized enzymatic dipoles. This desolvation penalty for transfer to an active site lacking the hydrogen-bond donors in the oxyanion hole is depicted by the free energy profiles of Figures 11A. The penalty becomes larger as charge localization increases in the transition state or as the phenolate pK _a_ increases in our experimental test system.

Next we consider the effects from adding in the hydrogen-bond donors present in the oxyanion hole ( Figure 13B). The shortening of the oxyanion hole hydrogen bonds partially compensates for the desolvation penalty by delocalizing the electron density of the oxyanion—the greater the charge localization on the free phenolate, the greater the bond shortening and compensation. This bond shortening, along with the increased electrostatic interaction with the hydrogen-bond donors with increasing charge, would be expected to contribute to the enthalpy of binding and is presumably responsible for some of the large increase in −ΔH with higher p *K*_a_ phenolates (ΔΔH = −2.0 kcal/mol/p *K*_a_ unit; Figure 11A; [ 66, 83, 84]). Nevertheless, differential active site rearrangements upon binding of phenolates with different degrees of charge localization may also contribute to the observed ΔΔH, and such active site rearrangements represent the final component of the model.

The change in the entropy of binding as the phenolate p *K*_a_ increases, Δ(TΔS), is very large (2.0 ± 0.3 kcal/mol/p *K*_a_ unit or 3.6 kcal/mol over the fluorophenolate series; Figure 11B), much larger than expected for the loss of mobility of a bound phenolate ion with successively shorter hydrogen bonds and correspondingly restricted motion. For example, the change in entropy to fully restrict a bond rotation, in the absence of accompanying contributions from rearrangements of the surroundings, is only 0.9–1.5 kcal/mol at 25 °C [ 1]. Thus, the large observed change in the entropy of phenolate binding cannot be accounted for by restriction of phenolate motion and suggests that the surroundings rearrange differentially in response to phenolates of increasing p *K*_a_. Such rearrangements would be expected for a dynamic enzymatic environment that is not fully preorganized to stabilize charge accumulation in the oxyanion hole. Enzymatic dipoles may both reorient and experience restricted motion due to the burden of “solvating” an increasingly localized negative charge within the active site. This is depicted in Figure 13 by the rearranged colored dipole bars and by the fewer surrounding motion lines. The energetic sum of these active site changes results in only a small net electrostatic contribution to catalysis.

The model summarized in Figure 13 predicts that if hydrogen bonds were prevented from shortening but negative charge still accumulated, the desolvation penalty would dominate the overall free energy change, leading to progressively weaker binding with increasing negative charge. Results with a series of sterically hindered substituted phenolates display this predicted weakening (unpublished data).

#### Perspective

The results herein indicate that hydrogen bonding in the active site of KSI provides little electrostatic advantage compared with water. Nonetheless, hydrogen-bonding groups are important for catalysis. Removal of a hydrogen-bond donor via site-directed mutagenesis can lead to an uncompensated desolvation penalty ( Figure 13A) and can thus lead to a situation in which catalysis is electrostatically inhibited. These mutations would also eliminate catalytic contributions from geometric complementarity of positioned active site hydrogen-bond donors and acceptors. Thus, active site hydrogen bonds may minimally enable a charged transition state to exist, without substantial energetic penalty, in an environment less polar than aqueous solvent. Enzymes may have taken advantage of this imperative to further fine-tune the positioning of active site hydrogen-bonding groups, providing optimal hydrogen bonding distances and angles for transition states but not bound substrates.

With KSI it was possible to isolate electrostatic features of the reaction using fluorophenolate transition state analogs and to test the role of electrostatic complementarity in catalysis. Additional systematic investigations will be required to test the role of electrostatics in other enzymes to broaden our understanding of electrostatic complementarity in catalysis. In many cases a ligand-based analysis as carried out for KSI may not be possible, and the incorporation of unnatural amino acids may provide a necessary alternative means to selectively and systematically perturb electrostatic features of active site interactions. Computation can also, in principle, provide a powerful probe of electrostatic effects by systematically modulating charge within an active site without affecting geometry. Selective tests of geometrical contributions to catalysis are also needed. Ultimately, synergistic experimental and computational approaches will be needed to provide consistent and mutually reinforcing descriptions of the energetic and structural underpinnings of electrostatic and geometrical complementarity in enzymatic catalysis.

## Materials and Methods

### Materials

All substituted phenols were of the highest purity commercially available (≥97%). Phenols were purchased from Sigma-Aldrich (St. Louis, Missouri, United States), except 3,4,5-trifluorophenol (Matrix Scientific, Columbia, South Carolina, United States); 3-fluoro-5-(trifluoromethyl)phenol (Oakwood Products, West Columbia, South Carolina, United States); and 2,3,5-trifluorophenol, 2,4,5-trifluorophenol, 2-fluorophenol and 3-ethylphenol (Acros Organics, Morris Planes, New Jersey, United States). Equilenin was from Steraloids (Newport, Rhode Island, United States); sodium 4,5-dihydroxynaphthalene-2,7-disulfonate and sodium-3-trimethylsilylpropionate-22,33- *d _4_* were from Sigma-Aldrich; Alexa Fluor 488 hydrazide was from Molecular Probes (Eugene, Oregon, United States); DMSO- *d _6_*, D _2_O and 4-nitrophenol- *d _4_* were from Cambridge Isotope Labs (Andover, Massachusetts, United States); 5 mm Shigemi symmetrical microtubes were from Shigemi (Allison Park, Pennsylvania, United States); and a standardized sodium hydroxide solution for calorimetry was from Mallinckrodt (Paris, Kentucky, United States). All buffers were prepared with reagent grade chemicals or better.

### Synthesis of EqA488, a fluorescent enzyme ligand

Alexa Fluor 488 hydrazide (0.1 mg, 0.2 mmoles, mixture of two isomers) was dissolved in 300 μL dimethylformamide. Equilenin (1.9 mg, 7 mmoles) was dissolved in 300 μL ethanol. The two solutions were mixed, glacial acetic acid was added (10% final volume), and the reaction was rotated at room temperature for 18 h. Solvent was then evaporated, and the hydrazone product ( Figure 9A) was resuspended in 100 mM triethylammonium acetate (pH 5.5), filtered, and purified by HPLC on a Vydac C18 analytical column (with a linear gradient from 100 mM triethylammonium acetate (pH 5.5) in water to 10 mM triethylammonium acetate in 90% acetonitrile), resolving the two isomers of EqA488. Both isomers had similar fluorescence properties and identical masses (calculated MW = 795.3, observed by electrospray mass spectrometry MW = 795.3) and the isomer with shorter retention time (EqA488–1) was further characterized.

Addition of 3 μM pKSI *^D40N^* to 30 nM EqA488–1 significantly quenched the fluorescence from the Alexa fluorophore (excitation at 490 nm, emission maximum at 515 nm), and addition of equilenin completely restored the solution fluorescence level (unpublished data). Addition of pKSI *^D40N^* to the unconjugated Alexa Fluor 488 hydrazide had no effect on the fluorescence (unpublished data). EqA488–1 bound with the hydroxyl group of equilenin ionized in the active site of pKSI *^D40N^* ( Figure 4D), as indicated by a shift in fluorescence excitation maximum from 330 to 342 nm detected at 400 nm emission, as observed with unmodified equilenin (unpublished data) [ 70].

### Mutagenesis

Plasmids encoding pKSI [ 129] and tKSI [ 130], which contain KSI inserted between the EcoRI and HinDIII sites of the pKK223–3 plasmid for the enzyme from P. putida and *C. testosteroni,* respectively, were gifts from Kwan Yong Choi and Ralph Pollack. QuikChange site-directed mutagenesis was used to introduce *D40N* mutations (Stratagene, La Jolla, California, United States), which were confirmed by sequencing miniprep DNA (Qiagen, Valencia, California, United States) from DH5-alpha cells on an ABI3100 capillary sequencer. For the P. putida enzyme, an additional mutation *(D34C)* was introduced. This mutation had less than a 2-fold effect on the activity of the WT enzyme (unpublished data).

### Expression and purification of KSI

KSI was purified by a previously described method, with minor modifications [ 131]. Briefly, a 20 mL overnight culture of BL21 cells (50 μg/mL carbenicillin) transformed with the plasmid encoding pKSI or tKSI was diluted into 2 L of LB supplemented with 50 μg/mL carbenicillin and 0.5 mM IPTG. Cells were grown for 8–12 h at 37 °C, harvested by centrifugation, resuspended in 40 mM potassium phosphate (pH 7.2), 1 mM EDTA, 2 mM DTT, and lysed by passage through a French pressure cell. The clarified cell lysate was passed over a deoxycholate-sepharose affinity column [ 129], washed with 0.4 M potassium phosphate (pH 7.2), 1 mM EDTA, 2 mM DTT, and then re-equilibrated with 40 mM potassium phosphate before being eluted with 40 mM potassium phosphate, 1 mM EDTA, 2 mM DTT, 50% ethanol. The enzyme was then further purified by gel filtration chromatography on a Superose-12 column in 40 mM potassium phosphate (pH 7.2), 1 mM EDTA, 2 mM DTT, and concentrated using Amicon Ultra centrifugal filter devices (Millipore, Billerica, Massachusetts, United States) with a 10 kDa molecular weight cutoff. Final purity was >99% as estimated based on a Coomasie-stained SDS-PAGE gel. Protein concentration was determined using the calculated molar extinction coefficient in 6 M guanidinium hydrochloride [ 132].

### Crystallization and X-ray data collection

pKSI *^D40N^* co-crystals with phenol, with the apparent symmetry of space group C222 _1_, were obtained by the method of hanging drop vapor diffusion. Crystallization conditions were obtained by mixing 2 μL of pKSI *^D40N^* at 23 mg/mL with 2 μL of a reservoir solution containing 1.4 M NH _4_SO _4_, 20 mM KH _2_PO _4_, 1 mM EDTA, 1 mM DTT, 5–6.5% 2-propanol, and 2–2.6 mM phenol (pH 7). Cube shaped crystals appeared after 5–7 days of incubation at room temperature. The average crystal size was 0.5 × 0.5 × 0.5 mm ^3^. Cryo-protection was achieved by first soaking crystals for 15 s in a solution of mother liquor diluted 1:1 with 2.9 M sodium malonate (pH 7), then transferring the crystals directly into the 2.9 M sodium malonate solution for 15 s. Crystals were rapidly frozen by submersion in liquid N _2_.

Data were collected in two passes with a Quantum 315 CCD detector at beamline 14-BMC of the Advanced Photon Source (Argonne National Laboratory, Argonne, Illinois, United States), with each pass consisting of 180° segments collected as 1° oscillations. The first pass, with an exposure time of 3 s, ensured the adequate collection of the highest resolution spots, while the second pass, with an exposure time of only 1 s, ensured that low resolution data was not overloaded during collection.

### X-ray crystallographic structure refinement

Due to a peculiar type of crystal disorder (unpublished data), data were reduced in spacegroup P1 instead of the apparent, and previously reported, spacegroup C222 _1_. An initial solution containing four copies of pKSI *^D40N^* in spacegroup P1 was determined using Phaser [ 133]. Initially, rigid body refinement, in which each of the four copies of the protein were treated as independent rigid bodies, was carried out in CNS [ 134], followed by conjugate-gradient coordinate minimization and restrained isotropic B-factor refinement. One round of water picking was also carried out in CNS. Further refinement and rebuilding and water picking was carried out in Coot [ 135] and Refmac5 [ 136], including anisotropic individual B-factor refinement (resulting in a 1.7% drop in *R _free_*, and a 3.4% decrease in *R _work_*).

### Determination of phenol p *K*_a_ values

Phenol p *K*_a_ values ( 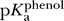) were taken from the literature [ 137] if available. Others were determined by spectral titration [ 138] at the absorbance maxima for the phenol and/or phenolate forms at 25 °C and an ionic strength of 100 mM, maintained with NaCl ( Table S4). The p *K*_a_ of equilenin was also determined by this method.

### ^1^H NMR spectroscopy

NMR experiments were performed using tKSI *^D40N^*, as the signal/noise of downfield peaks observed upon phenolate binding was larger for this enzyme than for the corresponding enzyme from *P. putida,* although nearly identical slopes were observed (unpublished data). ^1^H NMR experiments were carried out at the Stanford Magnetic Resonance Laboratory on 500-, 600-, and 800-MHz Varian ^UNITY^INOVA spectrometers running VNMR v6.1C (Varian, Palo Alto, California, United States) and equipped with 5-mm, triple resonance, gradient ^1^H ^13^C/ ^15^N probes under conditions similar to those described previously [ 69, 78]. NMR samples consisted of 0.3–2.0 mM tKSI *^D40N^* and 0.3–3.0 mM substituted phenol, in 40 mM potassium phosphate buffer (pH 7.2), 1 mM EDTA, 2 mM DTT, and 10% (v/v) DMSO- *d _6_* (which served as the deuterium lock solvent and prevented freezing at subzero temperatures) in 5 mm Shigemi symmetrical microtubes. Sample temperature was regulated at −3.0 ± 0.5 °C, verified using a neat methanol standard [ 139]. One-dimensional proton spectra were acquired using the 1331 binomial pulse sequence [ 140] to suppress the water signal, with a spectral width of 30 ppm (carrier frequency set on the water resonance) and an excitation maximum of 14–17 ppm. For each sample, a 90° pulse width was calibrated and data were collected with 32,000 points and a 1.9 s recycle delay for 512-5120 scans. The data were processed using a 10-Hz line broadening, and a baseline correction was applied over the downfield peaks of interest. Chemical shifts were referenced internally to the water resonance (5.1 ppm at −3 °C) [ 141] and externally to a sample of sodium-3-trimethylsilylpropionate-22,33- *d _4_* (0 ppm) in the same buffer conditions. Referencing was reproducible to ± 0.1 ppm for the same sample run multiple times as well as between samples from independent enzyme preparations.

Chemical shifts observed for both low and high p *K*_a_ phenolates were insensitive to changes in temperature (from −3 to 4 °C) or phenol concentration (from 0.3 to 3.0 mM). This invariance of chemical shift along with the low micromolar affinity observed for these substituted phenols binding to tKSI *^D40N^* under assay conditions (unpublished data) are consistent with slow exchange on the NMR timescale.

### Measuring the affinity of phenolates for KSI

The previous assay to measure the affinities of ligands for KSI relied on the quenching of enzyme fluorescence by bound ligand [ 69, 142]. This assay had several limitations due to the relatively weak emission of KSI in the ultraviolet (λ _max_ emission is ˜330 nm for pKSI and ˜310 nm for tKSI with 280 nm excitation). Most substituted phenols absorb in the UV, so the high concentrations of phenol needed to saturate the enzyme can lead to inner filter effects in which light is absorbed by the phenol rather than exciting the enzyme, giving apparent quenching of KSI fluorescence without binding [ 143]. Furthermore, the fluorescence of phenols, which are present in much higher concentration than the enzyme, can dominate the observed fluorescence, leading to small signal-to-noise in binding experiments.

To overcome these limitations we developed a new fluorescence assay in which substituted phenol binding displaced EqA488–1, a ligand with absorbance and fluorescence far to the red of other ligands, from the active site of KSI *^D40N^* ( Figure 9). Indeed, the affinities of several phenolates, measured using this new assay, were significantly different from those reported previously (e.g., apparent affinities for tKSI *^D40N^* [pH 7]: 4-chlorophenol, 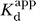 = 40 ± 7 μM versus 7.9 ± 1.3 μM and phenol, 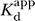 = 430 ± 80 μM versus 61 ± 10 μM for the new and previous assays, respectively [ 69]). The values obtained from the new fluorescence assay were independently confirmed by ITC for pKSI *^D40N^* (see Figure S2).

The new assay was carried out as follows. The affinity of EqA488–1 ( 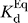) was determined by titration with enzyme, and the apparent affinity of the substituted phenol ( 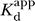) was then determined by displacement of EqA488–1. This apparent affinity was converted into the pH-independent affinity of the phenolate ( 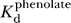), as depicted in the binding schemes of Figure 9D and described below. All measurements were made at 20 °C on a FluoroLog-3 spectrofluorometer (HORIBA Jobin Yvon, Edison, New Jersey, United States) with excitation at 480 nm, emission at 515 nm, and bandpass of 10 nm and 14.7 nm, respectively, using 0.1 nM EqA488–1 in a 45 μL microcuvette (Starna Cells, Atascadero, California, United States). Measurements were made using 10 mM buffer, 0.1 mM EDTA, and a constant ionic strength of 100 mM maintained with NaCl. pH-dependent measurements were made using acetate (pH 4.3–5.6), ADA (pH 5.6–7.1), HEPES (pH 6.9–8.1), and taurine (pH 8.1–10.2) buffers. In control experiments in which the concentration of EqA488–1 was reduced by half or the emission wavelength was changed by up to 5 nm, there was no effect on the determined affinity.

To determine the affinity for the reporter ligand EqA488–1 ( 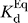 **)**, the enzyme concentration [E _T_] was varied above and below the K _d_ for the ligand (using individually prepared samples, typically 0.1–100 nM). After correcting for background fluorescence, the observed EqA488–1 fluorescence (F _obs_) as a function of enzyme concentration was fit to a quadratic binding isotherm ( Equation 1) using nonlinear regression analysis in Igor Pro (Wavemetrics, Lake Oswego, Oregon, United States), with the total EqA488–1 concentration [Eq _T_] held fixed and equal to the sum of bound and free EqA488–1, [Eq _bound_] and [Eq _free_] respectively.



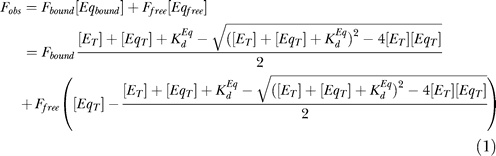



To determine the apparent affinities ( 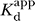) for the substituted phenol ligands by competition, EqA488–1 was displaced from the active site of KSI by the addition of the substituted phenol, giving an observed affinity ( 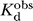) that has contributions from both the apparent affinity of the phenol under assay conditions ( 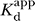) in the absence of EqA488–1 and from the initial concentration of the KSI•EqA488–1 complex ([E•Eq] in Figure 9D). Each substituted phenol was added to a mixture of enzyme (1-fold to 4-fold above 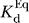) and EqA488–1 (0.1 nM). The observed fluorescence (F _obs_) as a function of phenol concentration [P _T_] was fit to a quadratic binding isotherm (Equation 2) to determine an observed affinity ( 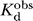). The value of 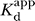 could then be determined from the known value of 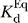, the total enzyme concentration [E _T_], and the observed affinity, 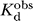, according to Equation 3 ( Figure 9D).



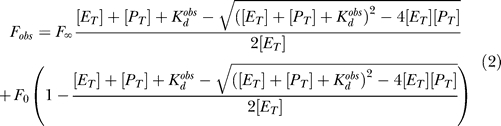





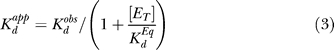



The pH-independent affinities for the substituted phenolates ( 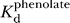 in Figure 9D) were determined as follows. The pH dependences of binding for several phenolates to pKSI *^D40N^* were measured. In all cases, the data fit well to Equation 4, with two p *K*_a_ values determining the apparent affinity. One corresponded to the p *K*_a_ of the phenol and the second to an enzyme p *K*_a_ of 5.5 (Text S2), presumably due to deprotonation of Asp103 [ 144]. Equation 4 was then used to determine the pH-independent affinity of each substituted phenolate from its p *K*_a_ and the apparent affinity determined experimentally (pH 8.0). Note that error in the determination of the enzyme p *K*_a_ will cancel out in a comparison of the relative affinities of substituted phenolates and therefore will not affect the conclusions drawn herein.



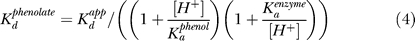



Data were plotted as affinity constants ( *K*_a_ values), which are simply the inverse of the dissociation constants ( *K*_d_ values). Error bars are the average deviation of 2–6 independent assays. Values of *K*_a_ for most phenolates were reproducible within 20% between assays.

### ITC

A displacement assay was used to measure the enthalpy of binding of substituted phenolates to pKSI *^D40N^* because many of the phenolates bound too weakly for direct titrations to be possible at reasonable enzyme concentrations. This approach is analogous to that described above for the determination of binding affinity via the fluorescence assay and has been described in detail previously [ 145, 146].

**Figure 14:**
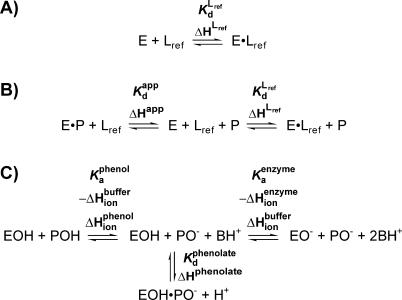
Determination of Thermodynamic Values for Phenolate Binding to KSI

Briefly, the affinity and enthalpy of binding of a high-affinity reference ligand (L _ref_) were first determined by direct titration of KSI ( Figure 14A) [ 147]. The titration was then repeated in the presence of a low affinity substituted phenol (P) of interest, such that KSI•P complexes must dissociate for KSI•L _ref_ to form. With 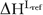 and 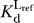 known, this measurement allows the determination of 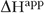 and 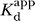 for the substituted phenol by direct fitting of the integrated heats ( Figure 14B). 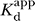 can be converted into the dissociation constant for the phenolate form of the ligand ( 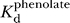) as described above for the binding assay ( Equation 4, Figure 14C).

The apparent enthalpy of binding measured for the substituted phenol ( 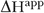) includes components from the enthalpies of ionization of the substituted phenol ( 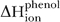), the enzyme ( 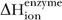), and the buffer ( 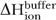), in addition to the desired enthalpy of binding of the phenolate to the enzyme ( 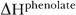), as depicted in Figure 14C. We are interested in the change in the binding enthalpy (ΔΔH) across a series of compounds rather than the enthalpy values for each compound. Thus, the constant contribution from the change in enthalpy associated with transfer of a proton from buffer to the enzyme ( 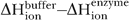, right side of Figure 14C) can be ignored for measurements conducted at a constant pH. Equation 5 allows comparison of the relative enthalpies of binding between phenolates (ΔH ^rel^) by correcting ΔH ^app^ for the enthalpy attributable to the fraction of phenol that must transfer its proton to buffer for binding to occur:



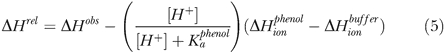



Enthalpies of ionization of phenols were measured using direct titration of phenol in 100 mM NaCl with 0.1 ± 0.0005 N NaOH and agreed well with available literature data ( Table S5) [ 148]. The enthalpy of ionization of HEPES, used to correct for proton transfer to buffer, is 5.02 kcal/mol [ 149].

Prior to ITC experiments, enzyme was dialyzed into buffer containing 10 mM HEPES (pH 7.2), 100 mM NaCl, 0.1 mM EDTA, 0.1 mM Tri(2-carboxyethyl)phosphine hydrochloride. All ligands were dissolved in the final dialysis buffer, and all samples were degassed under vacuum. ITC experiments were performed using a VP-ITC (MicroCal, Northampton, Massachusetts, United States) at 25 °C.

To determine the affinity and enthalpy for binding of the high affinity reference ligand, 3-isopropylphenol, 250–600 μM of the phenol was added in 8 μL increments to pKSI *^D40N^* (30–60 μM) in the calorimeter cell (1.4 mL) to a final concentration of 2–3× the enzyme concentration. The integrated heats were subtracted by the heats resulting from the addition of 3-isopropylphenol into buffer alone and were fit by nonlinear least-squares regression (Origin package, MicroCal), with the stoichiometry, association constant, and change of enthalpy of interaction as free variables.

In subsequent displacement experiments, 3-isopropylphenol was titrated into an enzyme solution with a constant concentration of another substituted phenol present in both the syringe and cell. After background subtraction, the data were fit by nonlinear least squares regression using a displacement model, with the stoichiometry, association constant, and change of enthalpy of interaction of the weak ligand as the free variables, and the association constant and change of enthalpy of the tight ligand fixed based on the values obtained from direct titration [ 146]. These data were then used to determine the affinity and ΔH ^rel^ for the phenolate form of the ligand as described above. Error bars represent the average deviation of two to five experiments at different concentrations of phenol. In some cases, phenols bound tightly enough that direct titration was possible, and these measurements gave results in good agreement with the displacement method ( Table S3).

## Supporting Information

Figure S1 Determination of Nonelectrostatic Effects on Phenolate Binding to KSI *^D40N^*
(A–B) Effects of alkyl substitutions at the *meta*- (red) and *para*- (blue) positions of the phenolate on affinity for pKSI *^D40N^* (A) and tKSI *^D40N^* (B).
(C) Effects of halogen substituents at the *meta*- (red) and *para*- (blue) positions of the phenolate on affinity for pKSI *^D40N^*.
(D) Dependence of the affinity of halogen-substituted phenolates from (C) on the molecular volume of the phenolate. Red and blue symbols indicate substitutions at the *meta-* and *para*-positions, respectively; unsubstituted phenol is represented in black. Linear fits to the *meta-* and *para*-position data (for iodine, bromine, and chlorine) suggest that there is no molecular volume effect for the fluorine substitutions (volume = 96 Å ^3^). Molecular volumes were calculated using VEGA WE ( http://www.ddl.unimi.it).
(57 KB PDF)

Figure S2 Comparison of Affinities of Fluorophenolates as Measured by Calorimetry (pH 7.2) (red) and Fluorescence Binding (pH 8.0) (blue) Slopes are 0.02 ± 0.04 and 0.11 ± 0.03, respectively.
(35 KB PDF)

Figure S3 Dependence of Affinity and Enthalpy of Binding of a Series of *ortho*-F Containing *para-* and *meta*-substituted Fluorophenolates for KSI *^D40N^* on p *K*_a_
Closed symbols show *ortho*-F containing fluorophenolates while open symbols are non- *ortho* substituted fluorophenolates from Figures 10 and 11 for comparison.
(A–B) The slopes for the affinity of the *ortho*-F containing series are −0.11 ± 0.09 for pKSI *^D40N^* (A) and 0.08 ± 0.06 for tKSI *^D40N^* (B).
(C) The slope for the enthalpy of binding of a series of *ortho*-F containing *para-* and *meta*-substituted fluorophenolates to pKSI *^D40N^* is −2.0 ± 0.4 kcal/mol/p *K*_a_ unit.
(48 KB PDF)

Table S1 Chemical Shifts of Downfield Peaks in tKSI *^D40N^*•phenolate Complexes
(25 KB DOC)

Table S2 Affinities of Phenolates to KSI *^D40N^* Determined by Fluorescence Binding
(25 KB DOC)

Table S3 Thermodynamics of Binding of Phenolates to pKSI *^D40N^* Determined by ITC
(25 KB DOC)

Table S4 p *K*_a_ Values for Phenols Used in This Work
(26 KB DOC)

Table S5 Heats of Ionization of Phenols (24 KB DOC)

Text S1 Changes in Charge Localization in the KSI Reaction (34 KB PDF)

Text S2 Substituted Phenols Bind as Phenolate Ions to KSI *^D40N^*
(316 KB PDF)

Text S3 Assignment of Downfield Peaks in ^1^H NMR Spectra of tKSI *^D40N^*•Phenolate Complexes
(788 KB PDF)

Text S4 Increased Charge Localization is Primarily Responsible for Shortened Hydrogen Bonds (39 KB PDF)

Text S5 Steric and Hydrophobic Effects of Phenolate Substituents (138 KB PDF)

Text S6 Calculation of Electrostatic Contribution to Catalysis (50 KB PDF)

### Accession Numbers

Accession numbers from the Swiss-Prot database ( http://www.expasy.org/sprot) are pKSI (P07445) and tKSI (P00947). pKSI *^D40N^*•phenolate X-ray structure coordinates have been deposited as 2B32 in the RCSB Protein Data Bank ( http://www.rcsb.org/pdb).

## Abbreviations

ITC: isothermal titration calorimetry
KSI: ketostero idisomerase
NMR: nuclear magnetic resonance
pKSI: KSI from *Pseudomonas putida*
tKSI: KSI from *Commamonas testosteroni*
