# Supplementary material for: Testing Electrostatic Complementarity in Enzyme Catalysis: Hydrogen Bonding in the Ketosteroid Isomerase Oxyanion Hole
Source: PLoS Biol. 2006 Mar 28;4(4):e99. doi: 10.1371/journal.pbio.0040099 (PMC1413570; doi:10.1371/journal.pbio.0040099)
Supplement: Supplementary file 1 [file pbio.0040099.sd001.pdf]

Text S1. Changes in Charge Localization in the KSI Reaction.

The use of substituted phenolates as a probe of electrostatic changes during the KSI reaction focuses on the degree of charge localization in the oxyanion hole, although charge distributions also change at other parts of the active site. The net amount of negative charge is conserved at the active site, as it is transferred from the carboxylate of Asp40 to the oxyanion of the dienolate intermediate in the course of reaction (Figure 4A). The charge on Asp40 in the ground state might be expected to lead to destabilization of the substrate relative to the transition state and intermediate, where the charge has been transferred to the oxyanion. However, binding of the substrate is essentially unaffected by the protonation state of Asp40 [1], indicating that this position is relatively insensitive to changes in charge localization. It remains possible that other smaller charge rearrangements away from the oxyanion hole are recognized for catalysis by KSI.

References

1. Pollack RM, Bantia S, Bounds PL, Koffman BM (1986) pH dependence of the kinetic parameters for 3-oxo-delta 5-steroid isomerase. Substrate catalysis and inhibition by (3s)-spiro[5 alpha- androstane-3,2'-oxiran]-17-one. Biochemistry 25: 1905-1911.
