# Supplementary material for: Testing Electrostatic Complementarity in Enzyme Catalysis: Hydrogen Bonding in the Ketosteroid Isomerase Oxyanion Hole
Source: PLoS Biol. 2006 Mar 28;4(4):e99. doi: 10.1371/journal.pbio.0040099 (PMC1413570; doi:10.1371/journal.pbio.0040099)
Supplement: Supplementary file 2 [file pbio.0040099.sd002.pdf]

Text S2. Substituted Phenols Bind as Phenolate Ions to KSI^{D40N}

To evaluate the role of electrostatic complementarity in the binding of a series of ligands to the oxyanion hole of KSI, it is necessary that the ligands bind ionized, so that changes in pK_a affect the degree of negative charge localized on the oxyanion. While the x-ray structure confirmed binding of the ligand in the oxyanion hole, it did not reveal whether the ligand bound ionized. Ligand ionization state was investigated to ensure that the series of compounds being used to mimic charge accumulation in an oxyanion hole were anionic.

The pH dependence of 4-cyanophenol binding to pKSI^{D40N} (Figure ST2A) indicated that the ionization of two groups affected binding. The data were consistent with one of these groups being the phenol (pK_a = 8.0). The pK_a value obtained from a fit to the data with one pK_a value held at 8.0 was 5.5 ± 0.1, suggesting an enzyme group that also affected binding[1]. An identical enzyme pK_a value was also obtained using tKSI^{D40N} (data not shown). Support for these assignments comes from the pH dependence for binding of other phenols. A global fit to data from binding of pentafluorophenol (pK_a = 5.7), 2,4,5-trifluorophenol (pK_a = 7.7), 4-cyanophenol (pK_a = 8.0) and 3-fluorophenol (pK_a = 9.3) in which the phenol pK_a values were held constant gave an identical enzyme pK_a of 5.4 ± 0.1 for pKSI^{D40N} (data not shown).

pH-dependence data indicate gain or loss of protons but cannot establish where protons are bound within a complex. Thus, the data were consistent with two models (Scheme ST2): either an enzyme group, tentatively assigned to Asp103, is protonated and

[1] If both pK_a values are allowed to vary in the curve fit, values of 8.3 ± 0.1 and 5.1 ± 0.1 are obtained.

the phenol deprotonated or the phenol is protonated and the enzyme group deprotonated in the complex.

Scheme ST2

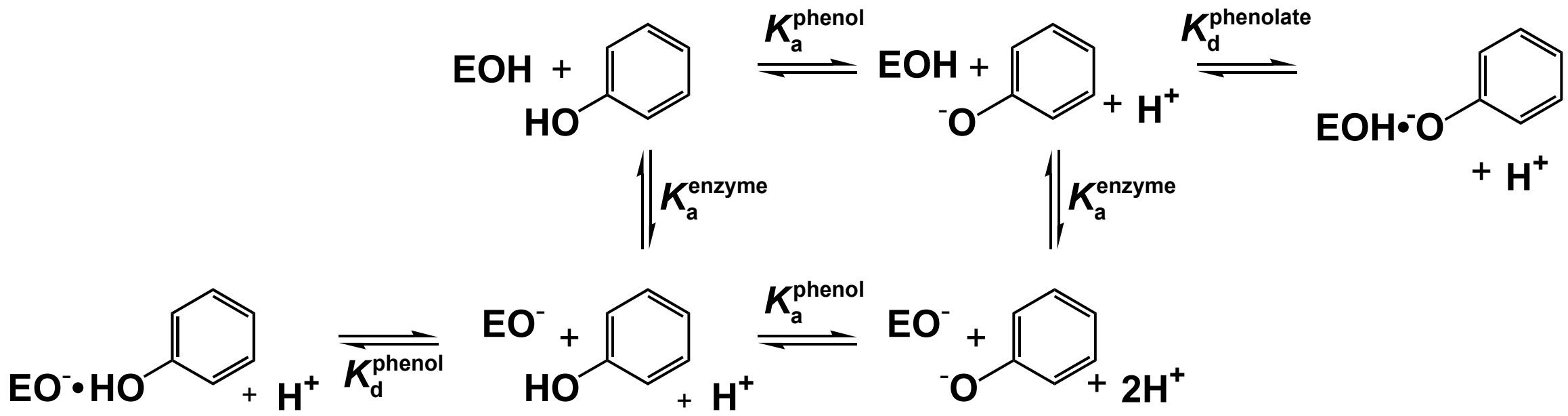

Previous experiments showed that phenols bound to KSI had absorbance spectra consistent with ionization to the phenolate at the active site [1]. We conducted further experiments to ensure that the substituted phenols bound as phenolate ions. These experiments (Figure ST2 and data not shown) utilized UV-VIS absorbance spectra and ^{19}F NMR spectra of a variety of phenols with pK_a values ranging from 5.4 to 9.8 bound to KSI^{D40N} and free in solution. In each case, the spectra of the phenol•enzyme complexes were compared to the neutral and ionized solution spectra of the same substituted phenols and suggested that the bound ligand was ionized in all cases. These results confirmed that the phenolate, not the enzyme, was ionized in the KSI•ligand complex, as previously suggested [1].

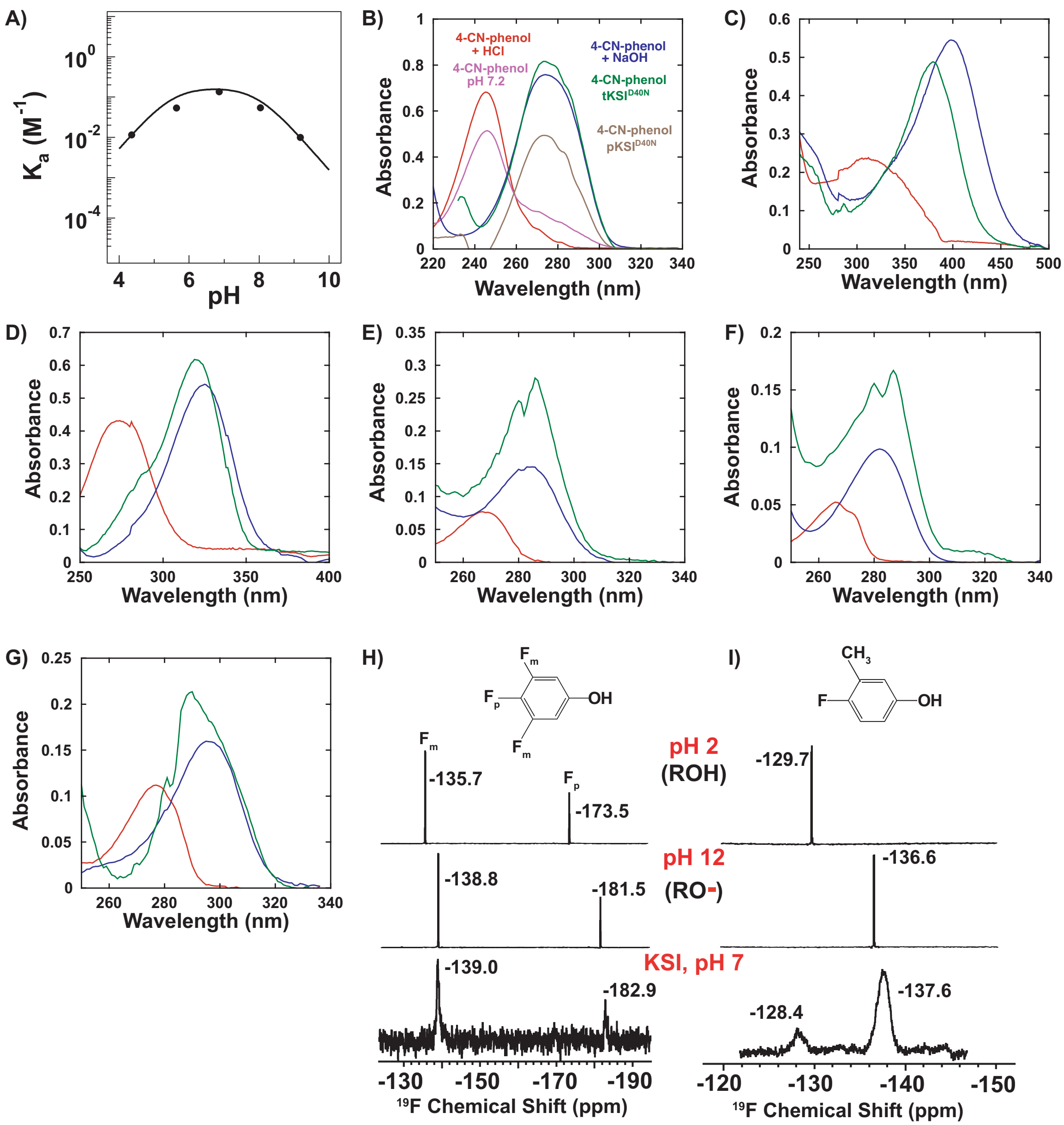

Figure ST2. Determination of ionization state of substituted phenol bound to the KSI active site. **A**) pH dependence of binding of 4-cyanophenol to pKSI^{D40N}. The pH-dependent apparent association constants were fit to Equation 4, giving an enzyme pK_a of 5.5 ± 0.1 and a K_d of 16 ± 2 nM with the phenol pK_a fixed at 8.0. The pH dependence of binding to tKSI^{D40N} gave an identical pK_a (data not shown). **B**) Absorbance spectra of 30 μM 4-cyanophenol free and bound to KSI^{D40N}. Absorbance maxima are at 245 nm for the

neutral phenol in 10 mM HCl (red) or 40 mM potassium phosphate, pH 7.2 (purple) and at 274 nm for the ionized phenolate in 10 mM NaOH (blue). In the presence of 60 μM $tKSI^{D40N}$ (green) or 40 μM $pKSI^{D40N}$ (brown) in 40 mM potassium phosphate, pH 7.2, the absorbance maximum is shifted to 274 nm, consistent with bound phenolate ion. **C-H**) Absorbance spectra of additional substituted phenols free and bound to $tKSI^{D40N}$ indicate that the ionized phenolate form binds to the enzyme. Spectra were measured in 10 mM HCl (red), 10 mM NaOH (blue) and bound to $tKSI^{D40N}$ (green) in 40 mM potassium phosphate, pH 7.2. In all cases, concentrations of enzyme and phenol were sufficient to give >95% bound phenol. **C**) 3,4-Dinitrophenol (pK_a = 5.4). **D**) 4-Acetylphenol (4′-hydroxyacetophenone) (pK_a = 8.1). **E**) 3,4,5-Trifluorophenol (pK_a = 8.2). **F**) 3-Fluorophenol (pK_a = 9.3). **G**) 4-Fluoro-3-methylphenol (pK_a = 9.8). **H**) ^{19}F NMR spectra of 3,4,5-trifluorophenol free and bound to $tKSI^{D40N}$. The bound species (in 40 mM potassium phosphate, pH 7.2) has peaks at -139.0 and -182.9 ppm, consistent with ionized phenolate at the active site. **I**) ^{19}F NMR spectra of 4-fluoro-3-methylphenol free and bound to $tKSI^{D40N}$. The bound species has a main peak at -137.6 ppm, consistent with ionized phenolate at the active site. The minor peak at -128.4 is consistent with the 6% free phenol present, determined by integration before baseline correction.

Methods

Absorbance Spectra of KSI•phenolate Complexes

Absorbance spectra of substituted phenols were acquired in a microcuvette on a Uvikon 9310 absorbance spectrophotometer. The spectra of 10 – 50 μM phenol in 40 mM potassium phosphate pH 7.2, 10 mM HCl (pH 2), and 10 mM NaOH (pH 12) were compared with the spectrum of the same concentration phenol in 40 mM potassium

phosphate pH 7.2 in the presence of 100 – 300 μM enzyme (phenol >95% bound, data not shown) after subtraction of the free enzyme spectrum.

^{19}F NMR Spectra of KSI•phenolate Complexes

^{19}F NMR spectra were acquired on a 500 MHz Varian ^{UNITY}INOVA NMR (operating at 470.23 MHz) running VNMR v6.1c and equipped with a 5 mm PFG switchable probe operating at ambient temperature (20 ˚C). 10% v/v D_2O served as the lock solvent and ^{19}F chemical shifts were referenced to an external standard of trifluoroacetic acid (-76.55 ppm relative to $CFCl_3$). Spectra for 20 mM free substituted phenols were obtained (12 scans) in 10 mM HCl (pH 2) and 10 mM NaOH (pH 12), conditions in which each phenol is protonated or ionized, respectively. Spectra were then obtained (240 - 4300 scans) for each phenol bound to KSI (40 mM potassium phosphate pH 7.2, 1 mM EDTA, 2 mM DTT, 1.5 mM enzyme, 1 mM phenol) under conditions in which the phenol was >90% bound (data not shown). The data were processed using a line broadening of 3 Hz for the free phenol and 10 Hz for the KSI•phenolate complexes and a baseline correction was applied over the observed peaks.

References

1. Petrounia IP, Pollack RM (1998) Substituent effects on the binding of phenols to the D38N mutant of 3-oxo-delta5-steroid isomerase. A probe for the nature of hydrogen bonding to the intermediate. Biochemistry 37: 700-705.
