# Supplementary material for: Testing Electrostatic Complementarity in Enzyme Catalysis: Hydrogen Bonding in the Ketosteroid Isomerase Oxyanion Hole
Source: PLoS Biol. 2006 Mar 28;4(4):e99. doi: 10.1371/journal.pbio.0040099 (PMC1413570; doi:10.1371/journal.pbio.0040099)
Supplement: Supplementary file 3 [file pbio.0040099.sd003.pdf]

Text S3. Assignment of Downfield Peaks in ^{1}H NMR Spectra of $tKSI^{D40N}$•Phenolate Complexes

Phenolates bind in the oxyanion hole of KSI and accept short hydrogen bonds from Tyr16 and Asp103 (Figure 7). The one-dimensional ^{1}H NMR spectrum of unliganded $tKSI^{D40N}$ enzyme displays a single peak at ~13 ppm in the far downfield (>11 ppm) region of the spectrum. Upon phenolate binding, two new downfield peaks at >14 ppm appear and move progressively downfield as the pK_a of the bound phenolate increases (Figure 8A). The appearance of these two peaks upon phenolate binding and their sensitivity to phenolate pK_a is readily accounted for by a model in which the two peaks arise from the hydrogen atoms in the two oxyanion hole hydrogen bonds donated by Tyr16 and Asp103 to the bound phenolate. This assignment was tested and supported by 2D ^{1}H NOESY NMR experiments that identify hydrogen atoms that are close in space, comparison to model compound chemical shifts, and mutations of the oxyanion hole residues.

NOE cross-peaks are expected in a ^{1}H NOESY spectrum between resonances for protons that are close in space, within ~5 Å [1]. Our assignment predicts that NOE cross-peaks should appear in a NOESY spectrum between the two downfield peaks and upfield resonances that correspond to the ring protons of a phenolate bound to $tKSI^{D40N}$. In the ^{1}H NOESY spectrum of $tKSI^{D40N}$ bound to 4-nitrophenolate (pK_a 7.1) strong NOE cross-peaks were observed between downfield peaks at 15.1 and 14.8 ppm and several overlapping upfield peaks in the aromatic region of 6.5-7.5 ppm, as well as to a single peak at 7.8 ppm (Figure ST3A). To determine whether any of these cross-peaks were due to the 4-nitrophenolate ring protons, which resonate at approximately 7 (*ortho*) and 8

(*meta*) ppm (data not shown), we next acquired an identical NOESY spectrum using 4-nitrophenolate-d_4 in which the ring hydrogen atoms were replaced with deuterium. For both downfield peaks, the cross-peaks at 7.8 and 7.1 ppm disappeared, while the rest of the spectrum was unchanged (Figure ST3B). This result indicated that the peaks at 7.8 and 7.1 ppm in the 4-nitrophenolate NOESY spectrum represented the phenolate ring protons. Observation of an NOE between the ring protons of phenolate and the protons giving rise to the downfield peaks in the tKSI^{D40N}•4-nitrophenolate spectrum confirmed that these protons are near in space. Peaks with chemical shifts >14 ppm generally arise from protons involved in hydrogen bonds [1]. Based on the pKSI^{D40N}•phenolate crystal structure, three hydrogen bonds are within ~5 Å of the phenolate ring protons: that between Tyr16 and the phenolate oxyanion, that between Asp103 and the phenolate, and that between Tyr57 and Tyr16 (Figure ST3C).

The observation that the chemical shifts of downfield peaks in KSI spectra vary systematically with phenolate pK_a strongly suggests that these peaks arise from hydrogen bonds formed directly to the phenolate, *i.e.*, the Tyr16-phenolate and Asp103-phenolate hydrogen bonds, rather than the Tyr57-Tyr16 hydrogen bond. Assignment to the Tyr57-Tyr16 hydrogen bonded proton is further disfavored as both Tyr residues are likely to be protonated. Far downfield (>14 ppm) O-H--O hydrogen bond chemical shifts are typically observed for oxyanionic but not neutral hydroxylic hydrogen bond acceptors [2]. This behavior is exemplified in the following examples of short neutral and oxyanionic hydrogen bonds in small molecules. The neutral hydrogen bond formed by sodium 4,5-dihydroxynaphthalene-2,7-disulfonate has an O-O distance of ~2.55 Å and serves as a model for a short tyrosine-tyrosine hydrogen bond [3 and analysis of

Cambridge Structural Database not shown]. The hydroxyl proton in this hydrogen bond (Scheme ST3A) resonates with a chemical shift of 10.9 ppm in DMSO (unpublished results). Treatment with a molar equivalent of triethylamine deprotonates one of the hydroxyl groups to generate an oxyanionic intramolecular hydrogen bond (Scheme ST3B) in which the bridging proton resonates at 17.8 ppm [4 and unpublished results]. This general behavior is also displayed by substituted salicylic acids, in which the hydrogen bonded hydroxyl proton resonates at ~11.5 ppm in the neutral compound and >14 ppm in the monoanion [5]. In summary, the hydrogen bond behavior displayed by these model compounds is consistent with the Tyr57-Tyr16 hydrogen bonded proton resonating with a chemical shift <14 ppm and therefore strongly suggests that this proton does not give rise to either of the two downfield peaks observed in the tKSI^{D40N}•phenolate spectra.

Scheme SR2.

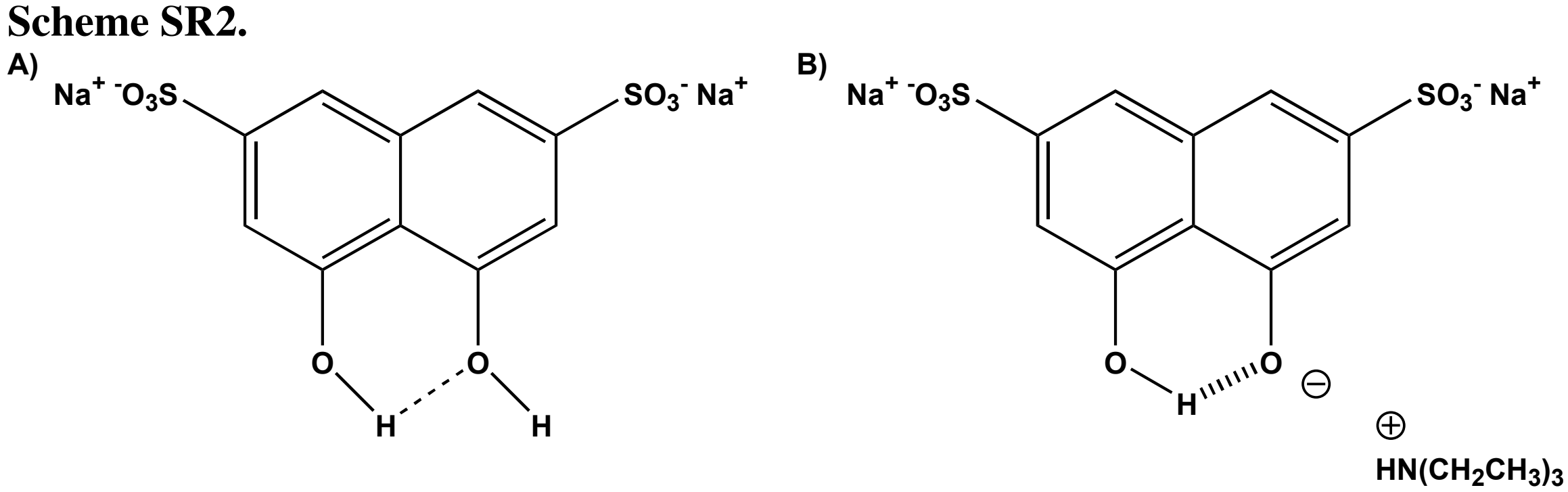

From the results above, we conclude that the two downfield peaks in the one-dimensional spectra of tKSI^{D40N}•phenolate complexes arise from hydrogen bonds donated directly to the phenolate oxygen, *i.e.*, the Tyr16-phenolate and Asp103-phenolate hydrogen bonds. It remains possible that the two peaks do not arise from two different hydrogen bonds to the phenolate, but from one hydrogen bond in slow exchange between

two conformations. Whether the two peaks represent two hydrogen bonds or one hydrogen bond in two conformations does not affect the interpretations or conclusions made in this paper. However, the effects of oxyanion hole mutations on the observed ^1H NMR spectrum of $tKSI^{D40N}$•3,4-dinitrophenolate (pK_a 5.4) support assignment of the two peaks to the Tyr16-phenolate and Asp103-phenolate hydrogen bonds. Only one downfield peak is observed upon mutation of Asp103 (to Asn, Leu, or Ala) or Tyr16 (to Phe), the simplest result expected for removing one but not both of the oxyanion hole hydrogen bond donors (unpublished results).

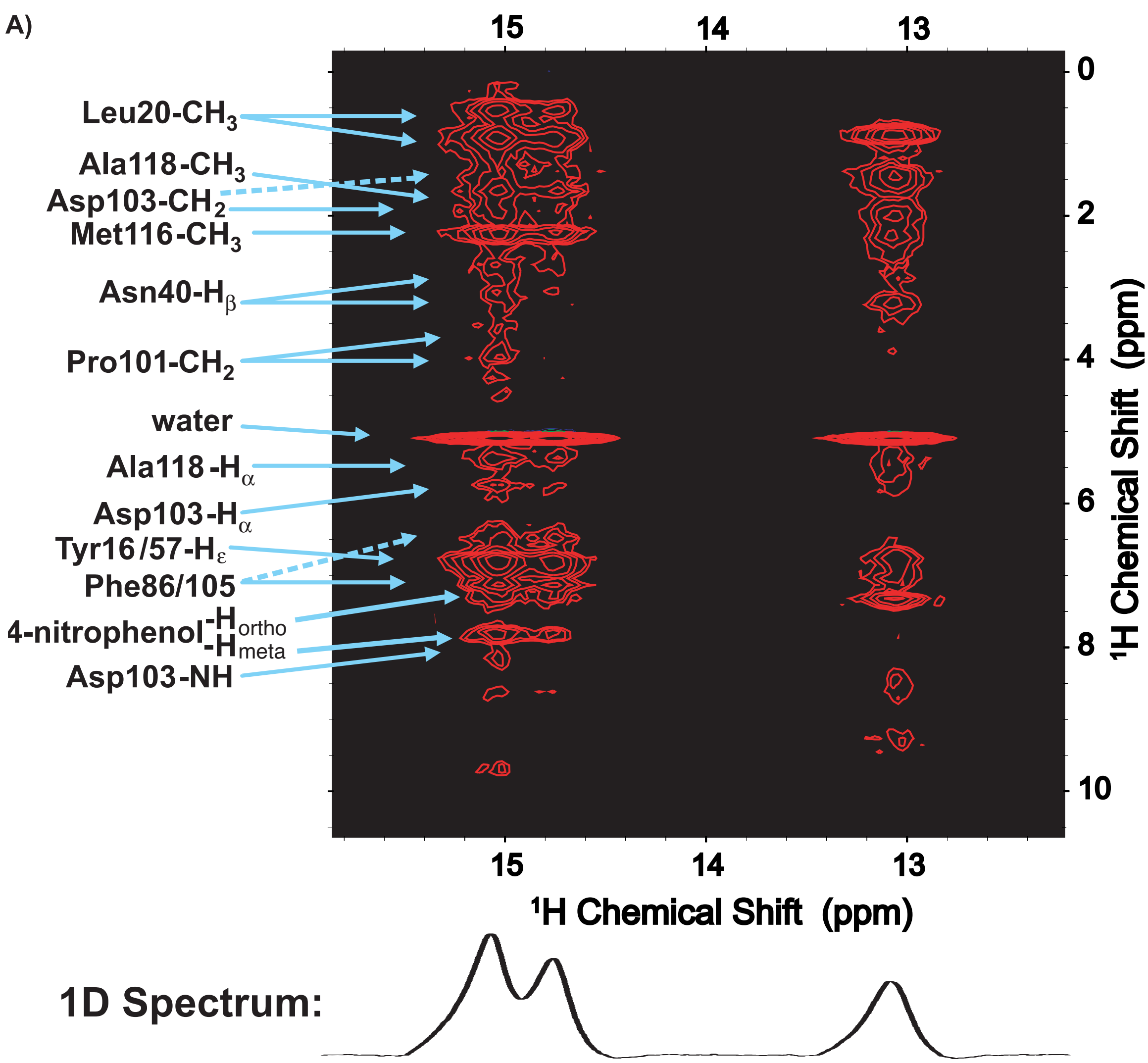

B)

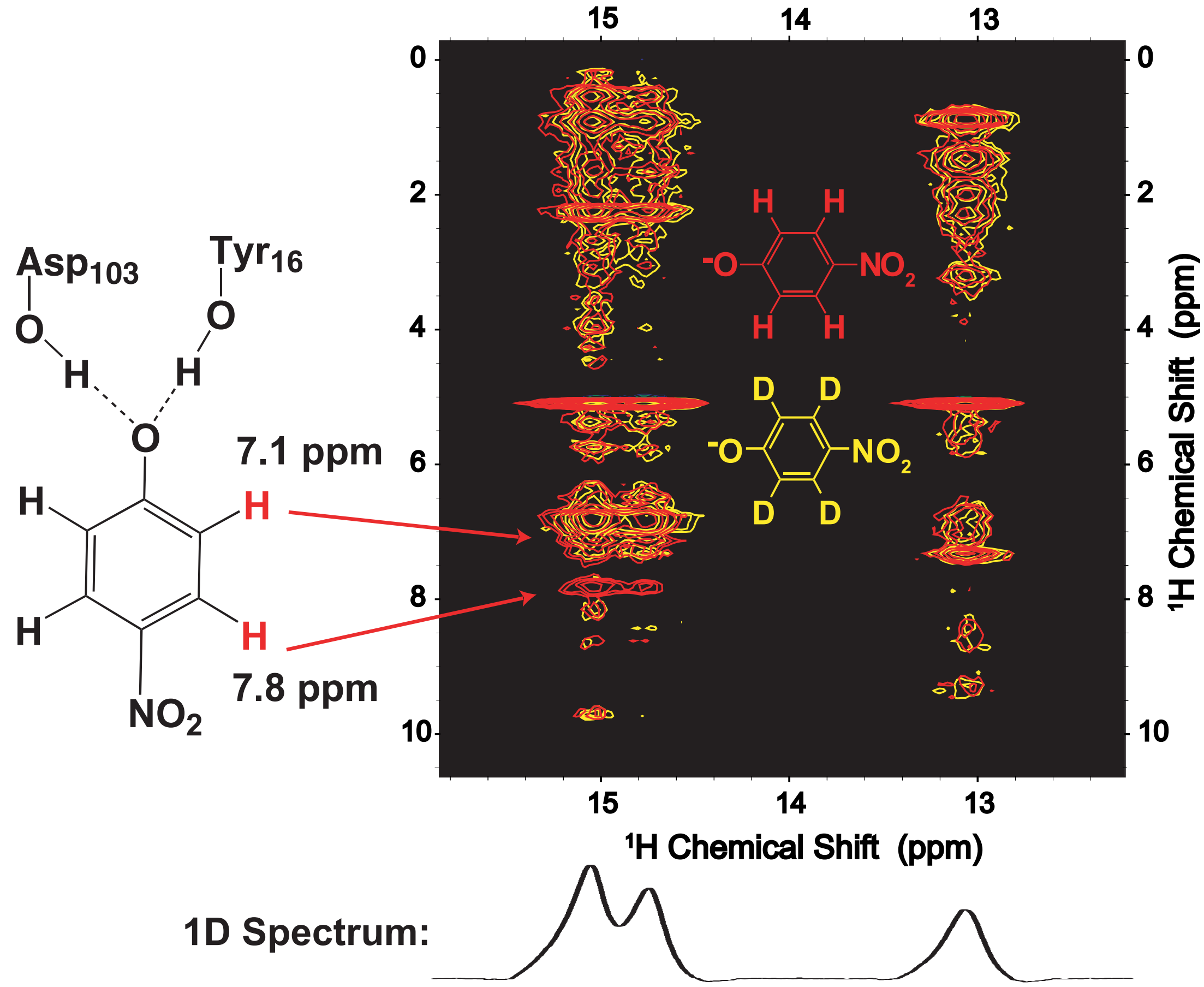

Asp103
Tyr16
O
H
H
O
O
H
H
H
H
NO2
7.1 ppm
7.8 ppm
15
14
13
0
2
4
6
8
10
-O
NO2
H
D
1H Chemical Shift (ppm)
1D Spectrum:

C)

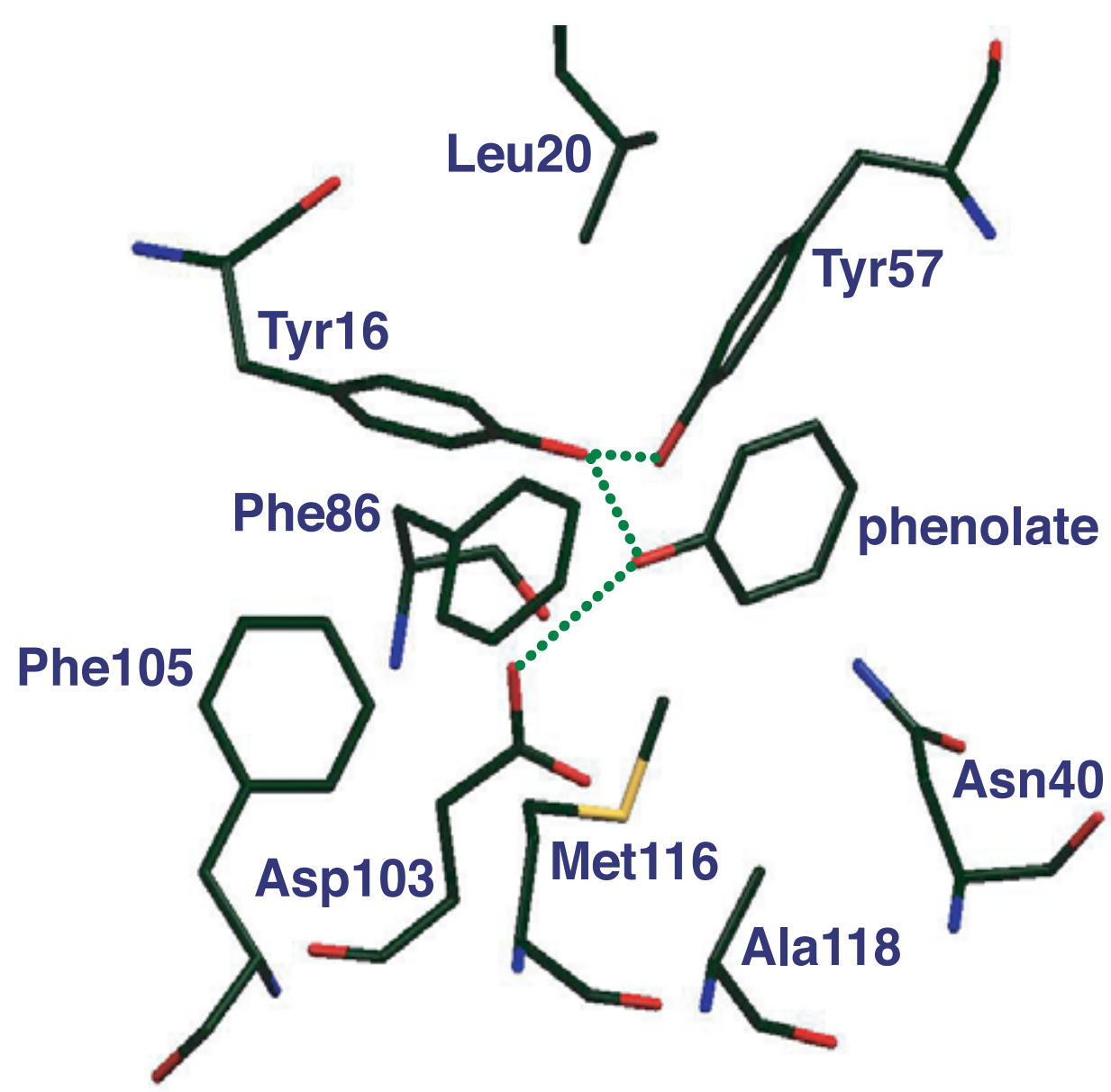

Leu20
Tyr57
Tyr16
Phe86
phenolate
Phe105
Asn40
Asp103
Met116
Ala118

Figure ST3. Assignment of downfield ^{1}H NMR chemical shifts to hydrogen bonds in the oxyanion hole. **A)** ^{1}H-^{1}H NOESY spectrum of tKSI^{D40N}•4-nitrophenolate. NOE cross-peaks to downfield resonances at 14.8 and 15.1 ppm have been assigned to oxyanion hole residues based on the ^{1}H assignments from the solution structure of tKSI$^{Y57F/Y92F}$ (pKSI numbering) [6]. **B)** Overlay of ^{1}H-^{1}H NOESY spectra of tKSI^{D40N}•4-nitrophenolate (red) and tKSI^{D40N}•4-nitrophenolate-d_4 (yellow). **C)** Crystal structure of pKSI^{D40N}•phenolate complex showing tKSI^{D40N} residues within 5 Å of the phenolate oxygen. Residues are labeled according to their identity in tKSI^{D40N} but with pKSI numbering. Green dots represent putative hydrogen bonds.

Methods

^{1}H-^{1}H NOESY Spectra of tKSI^{D40N}•4-nitrophenolate Complexes

Two dimensional ^{1}H-^{1}H NOESY spectra of tKSI^{D40N}•4-nitrophenolate complexes (2.0 mM enzyme, 5.0 mM 4-nitrophenol or 4-nitrophenol-d_4, 40 mM potassium phosphate pH 7.2, 1 mM EDTA, 2 mM DTT, 10% v/v DMSO-d_6, in 5 mm Shigemi microtubes) were acquired on the 800 MHz Varian ^{UNITY}INOVA NMR spectrometer at the Stanford Magnetic Resonance Laboratory running VNMR v6.1C (Varian, Inc.). Temperature was controlled at -3.0 ± 0.5 ˚C, and chemical shifts were referenced internally to the water resonance (5.1 ppm at -3.0 ˚C) as described in the main Methods. The SS-NOESY pulse sequence [7] (50 millisecond mixing time) was utilized for minimal saturation of protons exchanging with bulk water. Data were acquired over 23.8 ppm spectral widths of 2048 data points, 1.55 second recycle delay, and 64 scans per t_1 increment over 256 increments. The total time for each experiment was 16 hours. Spectra

were processed using shifted, squared sine bell window functions in both dimensions and one-fold zero filling in the t_1 dimension. Further analyses of the NOESY spectra were performed with the program SPARKY [8].

References

1. Cavanagh J, Fairbrother WJ, Palmer AG, Skelton NJ (1996) Protein NMR spectroscopy: Principles and practice. San Diego: Academic Press. 587 p.

2. Kumar GA, McAllister MA (1998) Theoretical investigation of the relationship between proton NMR chemical shift and hydrogen bond strength. J Org Chem 63: 6968-6972.

3. Mildvan AS, Massiah MA, Harris TK, Marks GT, Harrison DHT, et al. (2002) Short, strong hydrogen bonds on enzymes: NMR and mechanistic studies. Journal of Molecular Structure 615: 163-175.

4. Zhao Q, Abeygunawardana C, Talalay P, Mildvan AS (1996) NMR evidence for the participation of a low-barrier hydrogen bond in the mechanism of delta 5-3-ketosteroid isomerase. Proc Natl Acad Sci U S A 93: 8220-8224.

5. Shan SO, Herschlag D (1996) The change in hydrogen bond strength accompanying charge rearrangement: Implications for enzymatic catalysis. Proc Natl Acad Sci U S A 93: 14474-14479.

6. Massiah MA, Abeygunawardana C, Gittis AG, Mildvan AS (1998) Solution structure of delta 5-3-ketosteroid isomerase complexed with the steroid 19-nortestosterone hemisuccinate. Biochemistry 37: 14701-14712.

7. Smallcombe SH (1993) Solvent suppression with symmetrically-shifted pulses. J Am Chem Soc 115: 4776-4785.

8. Goddard TD, Kneller DG (2004) Sparky 3. University of California, San Francisco.
