# Supplementary material for: Testing Electrostatic Complementarity in Enzyme Catalysis: Hydrogen Bonding in the Ketosteroid Isomerase Oxyanion Hole
Source: PLoS Biol. 2006 Mar 28;4(4):e99. doi: 10.1371/journal.pbio.0040099 (PMC1413570; doi:10.1371/journal.pbio.0040099)
Supplement: Supplementary file 4 [file pbio.0040099.sd004.pdf]

Text S4. Increased Charge Localization is Primarily Responsible for Shortened Hydrogen Bonds.

As the charge localization on phenolate oxygens increased, KSI•phenolate hydrogen bond lengths shortened, as determined by NMR (Figure 8). An increase in the pK_a and charge localization of the phenolate oxygen is expected to be accompanied by a small increase in the phenolate C-O distance. A change of ~0.009 Å/pK_a unit is obtained from C-O distances observed in x-ray structures of substituted phenolates in the Cambridge Structural Database (analysis not shown). This change is ~3 fold less than the change in KSI•phenolate hydrogen bond lengths determined by the NMR measurements, suggesting that increased charge localization is primarily responsible for the shortened hydrogen bonds. Nevertheless, lengthening of the C-O bond in the course of reaction could contribute to transition state stabilization in the enzymatic active site by allowing the oxyanion to extend deeper into the oxyanion hole.
