# Supplementary material for: Testing Electrostatic Complementarity in Enzyme Catalysis: Hydrogen Bonding in the Ketosteroid Isomerase Oxyanion Hole
Source: PLoS Biol. 2006 Mar 28;4(4):e99. doi: 10.1371/journal.pbio.0040099 (PMC1413570; doi:10.1371/journal.pbio.0040099)
Supplement: Supplementary file 5 [file pbio.0040099.sd005.pdf]

Text S5. Steric and Hydrophobic Effects of Phenolate Substituents

Substituents on phenolates, introduced to vary the pK_a, can potentially interact with KSI directly. Thus, a change in binding affinity could arise from electrostatic or direct binding effects. As the binding pocket of KSI is hydrophobic we refer to such direct effects as "hydrophobic" below for simplicity. We set out to test the role of hydrophobic effects on binding in an attempt to find a series of compounds without hydrophobic effects, whose pK_a changes would result in only electrostatic changes to binding.

Petrounia and Pollack showed that the affinity of a variety of substituted phenolates for $tKSI^{D40N}$ could be fit using a two-parameter equation in which affinity depended on both electrostatics (pK_a) and hydrophobicity (log P) [1]. However, the binding assay that they employed had several limitations, as discussed in the Methods. Therefore, we designed a new binding assay to overcome these limitations, as described in the main body of the paper (Figure 9) and set out to directly test whether hydrophobicity affected binding.

The previously reported correlation predicted that for a series of substituted phenolates of constant pK_a but varying hydrophobicity (log P), the slope of a plot of the log of the association constant versus log P would be 0.6, the value obtained from a two-component fit to the prior data [1]. The affinities of a series of alkyl-substituted phenolates, which varied in hydrophobicity but not substantially in pK_a (range = 9.9-10.2), were examined. As the hydrophobicity and size of the substituents were increased, binding became tighter. The slope of the correlation between the affinity (log K_a) and the hydrophobicity (log P) was 1.5 ± 0.1, substantially larger than the previously reported

value of 0.6 [1] (Figure ST5). This difference indicates that the previous data cannot be used to separate the effects of hydrophobicity from the effects of electrostatics. We therefore set out to find a series of compounds without significant hydrophobic effects on binding that would allow an analysis of electrostatic effects, as described in the Results and Discussion.

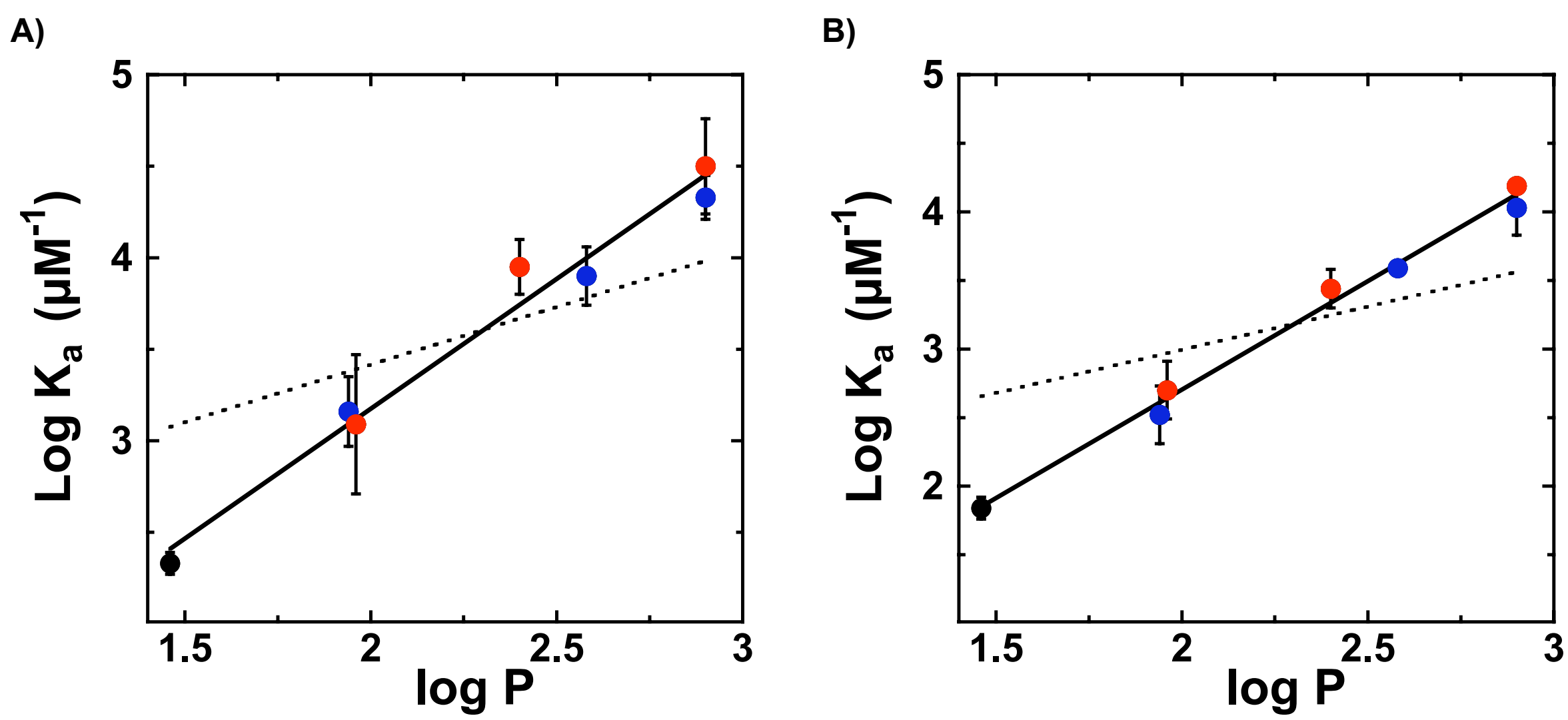

Figure ST5. Determination of non-elecrostatic effects on phenolate binding to pKSI^{D40N} (**A**) and tKSI^{D40N} (**B**). Dependence of affinity of alkyl-substituted phenolates on log P, the water-octanol partitioning coefficient for the phenol [2,3]. Red and blue symbols indicate substitutions at the *meta*- and *para*-positions respectively; unsubstituted phenol is represented in black. Compounds are phenol (pK_a = 10.0), 3-methylphenol (pK_a = 10.1), 4-methylphenol (pK_a = 10.2), 3-ethylphenol (pK_a = 9.9), 4-ethylphenol (pK_a = 10.0), 3-isopropylphenol (pK_a = 9.9), and 4-isopropylphenol (pK_a = 10.0). The solid lines are linear least squares fits, giving slopes of 1.4 ± 0.1 and 1.6 ± 0.1, respectively (R^2 = 0.97, 0.99). The dotted lines are fits to a line of slope 0.63, the value obtained from the

previous two-parameter correlation for binding of a series of phenols that varied in both pK_a and hydrophobicity [1], and give R^2 coefficients of 0.67 and 0.63, respectively.

References

1. Petrounia IP, Pollack RM (1998) Substituent effects on the binding of phenols to the D38N mutant of 3-oxo-delta5-steroid isomerase. A probe for the nature of hydrogen bonding to the intermediate. Biochemistry 37: 700-705.

2. Hansch C, Leo A (1987) Pomona college medicinal chemistry project. Log P Database. Claremont, CA.

3. Fujita T, Iwasa J, Hansch C (1964) A new substituent constant derived from partition coeffecients. J Am Chem Soc 86: 5175-5180.
