# Supplementary material for: Testing Electrostatic Complementarity in Enzyme Catalysis: Hydrogen Bonding in the Ketosteroid Isomerase Oxyanion Hole
Source: PLoS Biol. 2006 Mar 28;4(4):e99. doi: 10.1371/journal.pbio.0040099 (PMC1413570; doi:10.1371/journal.pbio.0040099)
Supplement: Supplementary file 6 [file pbio.0040099.sd006.pdf]

Text S6. Calculation of Electrostatic Contribution to Catalysis.

The slope of the dependence of phenolate affinity on pK_a (Figure 10) can be used to calculate the total electrostatic contribution to catalysis, which equals $10^{slope*(pK_a^{TS}-pK_a^{GS})}$. The maximal p$K_a$ difference between the ground state and the transition state is 16. The average slope (based on all four correlations above) was 0.05 ± 0.10, giving a maximum slope of 0.15, which would translate into a 250-fold rate enhancement. Similarly, the minimum slope of -0.05 would translate into a 6-fold barrier to catalysis. Also, the phenolates probed in Figure 10 spanned only ~2 pK_a units, so any nonlinearity over the larger pK_a difference between the ground state and transition state could lead to larger or smaller effects, although there was no indication of a significant change in slope over the extended pK_a range investigated with the *ortho*-F substituted phenolates (Figure S3).
