# Supplementary material for: Testing Electrostatic Complementarity in Enzyme Catalysis: Hydrogen Bonding in the Ketosteroid Isomerase Oxyanion Hole
Source: PLoS Biol. 2006 Mar 28;4(4):e99. doi: 10.1371/journal.pbio.0040099 (PMC1413570; doi:10.1371/journal.pbio.0040099)
Supplement: Supplementary file 7 [file pbio.0040099.sg001.pdf]

Figure S1

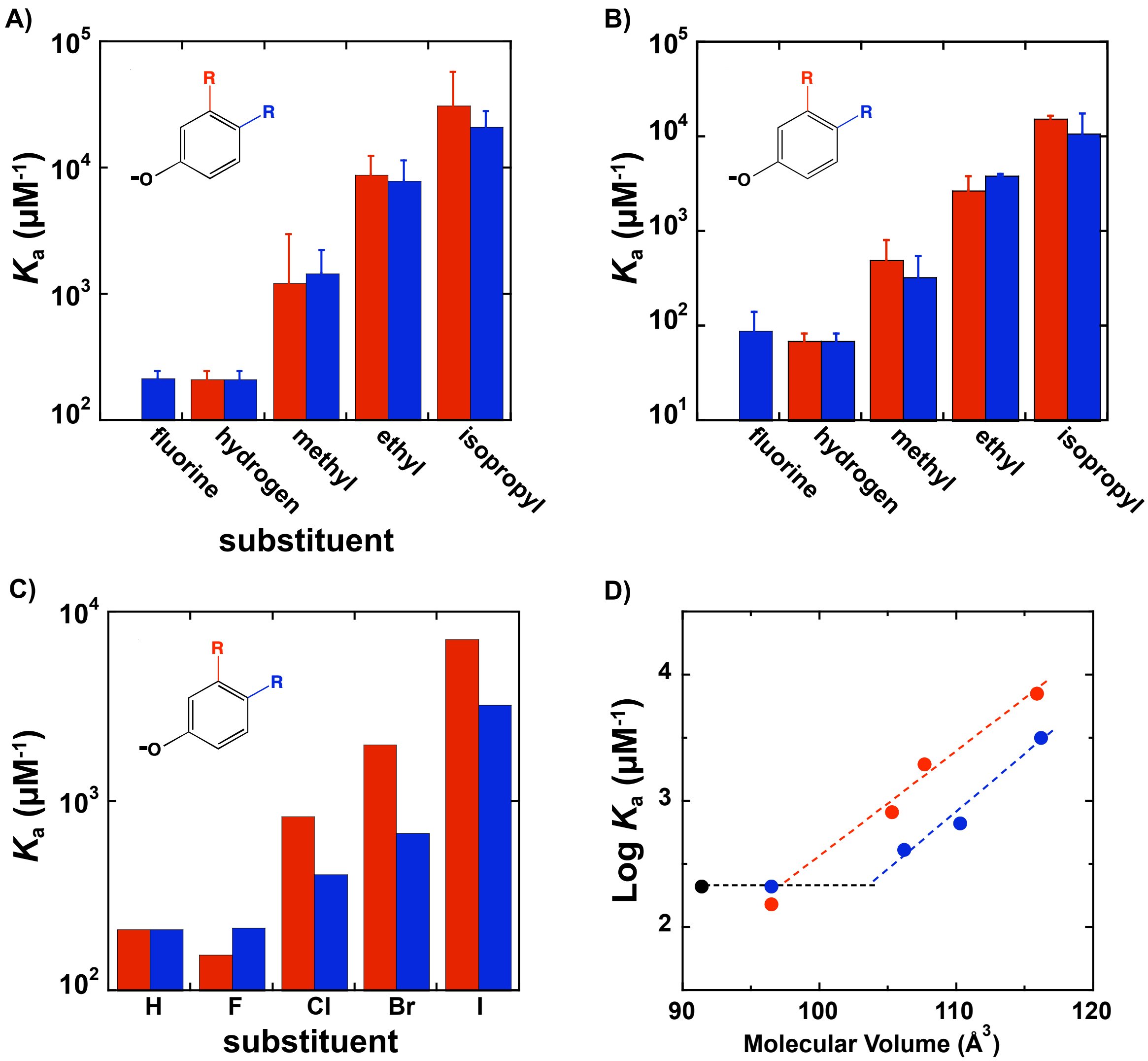

A)
B)
C)
D)
K_a (μM^{-1})
Log K_a (μM^{-1})
R
R
-O
fluorine
hydrogen
methyl
ethyl
isopropyl
H
F
Cl
Br
I
substituent
Molecular Volume (Å^3)
