# Supplementary material for: Testing Electrostatic Complementarity in Enzyme Catalysis: Hydrogen Bonding in the Ketosteroid Isomerase Oxyanion Hole
Source: PLoS Biol. 2006 Mar 28;4(4):e99. doi: 10.1371/journal.pbio.0040099 (PMC1413570; doi:10.1371/journal.pbio.0040099)
Supplement: Supplementary file 10 [file pbio.0040099.st001.doc]

**Table S1**. Chemical shifts of downfield peaks in tKSI^D40N^•phenolate complexes.

| Phenolate | **p*K*_a_** | **δ_1_ (ppm)** | **δ_2_ (ppm)** |
| --- | --- | --- | --- |
| 3,4-(NO_2_)_2_ | 5.4 | 14.2 | 14.0 |
| 3-F-4-NO_2_ | 6.1 | 14.4 | 14.3 |
| 3-CF_3_-4-NO_2_ | 6.3 | 14.9 | 14.5 |
| 4-NO_2_ | 7.1 | 15.0 | 14.8 |
| 3-NO_2_-4-Cl | 7.8 | 15.8 | 15.4 |
| 4-CN | 8.0 | 15.6 | * |
| 4-acetyl | 8.1 | 16.0 | 15.4 |
| 3,5-Cl_2_ | 8.1 | 16.6 | 14.8 |
| 3-F-5-CF_3_ | 8.2 | 16.4 | 15.6 |
| 3,4,5-F_3_ | 8.2 | 16.3 | 14.9 |
| 3-NO_2_ | 8.4 | 16.4 | 15.5 |
| 3,4-Cl_2_ | 8.5 | 16.4 | 15.6 |
| 3-CN | 8.6 | 16.4 | 15.6 |
| 3-CF_3_ | 8.7 | 17.0 | 16.1 |
| 4-CF_3_ | 8.9 | 16.4 | 15.9 |
| 3-Cl | 9.0 | 17.1 | 15.6 |
| 3-Br | 9.1 | 17.0 | 15.6 |
| 3-I | 9.2 | 16.8 | 15.9 |
| 4-I | 9.3 | 16.7 | * |

*Only 1 downfield peak observed.
