# Supplementary material for: Testing Electrostatic Complementarity in Enzyme Catalysis: Hydrogen Bonding in the Ketosteroid Isomerase Oxyanion Hole
Source: PLoS Biol. 2006 Mar 28;4(4):e99. doi: 10.1371/journal.pbio.0040099 (PMC1413570; doi:10.1371/journal.pbio.0040099)
Supplement: Supplementary file 11 [file pbio.0040099.st002.doc]

**Table S2**. Affinities of phenolates to KSI^D40N^ determined by fluorescence binding.

|  |  | **pKSI^D40N^** | | **tKSI^D40N^** | |
| --- | --- | --- | --- | --- | --- |
| **Phenol** | **p*K*_a_** | $K_{d}^{\text{app}}$ **(µM)^a^** | ***K*_d_ (nM)^a^** | $K_{d}^{\text{app}}$ **(µM)^a^** | ***K*_d_ (nM)^a^** |
| 4-F | 10.0 | 133 (15) | 4.6 (0.5) | 320 (110) | 11 (4) |
| Phenol | 10.0 | 135 (18) | 4.7 (0.6) | 410 (70) | 14 (2) |
| 3-F | 9.3 | 41 (8) | 6.4 (1.3) | 110 (4) | 17.4 (0.6) |
| 3,4-F_2_ | 9.1 | 31 (8) | 7.0 (1.9) | 69 (9) | 16 (2) |
| 3,5-F_2_ | 8.4 | 8.4 (1.0) | 8.0 (0.9) | 20.0 (0.4) | 18.9 (0.3) |
| 3,4,5-F_3_ | 8.2 | 6.1 (0.5) | 6.8 (0.6) | 17 (3) | 19 (4) |

^a^$K_{d}^{\text{app}}$ is defined as the apparent affinity at pH 8. *K*_d_ is defined as the pH-independent affinity. Errors (in parenthesis) are average deviations of 2-4 replicate measurements. Error between replicates was typically <10%.
