# Supplementary material for: Testing Electrostatic Complementarity in Enzyme Catalysis: Hydrogen Bonding in the Ketosteroid Isomerase Oxyanion Hole
Source: PLoS Biol. 2006 Mar 28;4(4):e99. doi: 10.1371/journal.pbio.0040099 (PMC1413570; doi:10.1371/journal.pbio.0040099)
Supplement: Supplementary file 12 [file pbio.0040099.st003.doc]

**Table S3**. Thermodynamics of binding of phenolates to pKSI^D40N^ determined by ITC.

| **Phenolate** | **p*K*_a_** | **Assay type** | **∆G**  **(kcal/mol)^a^** | **∆H_rel_**  **(kcal/mol)^a^** |
| --- | --- | --- | --- | --- |
| 4-F | 10.0 | Displacement | -11.34 (0.02) | -9.8 (0.1) |
| Phenol | 10.0 | Displacement | -11.3 (0.1) | -9.5 (0.4) |
| 3-F | 9.3 | Displacement | -11.10 (0.05) | -8.3 (0.1) |
| 3,4-F_2_ | 9.1 | Displacement | -11.14 (0.01) | -8.53 (0.05) |
|  |  |  |  |  |
| 3,5-F_2_ | 8.4 | Displacement | -11.1 (0.2) | -6.7 (0.8) |
|  | 8.4 | Direct Titration | -11.23 (0.03) | -6.9 (0.1) |
|  |  |  |  |  |
| 3,4,5-F_3_ | 8.2 | Displacement | -11.1 (0.1) | -5.8 (0.3) |
|  | 8.2 | Direct Titration | -11.51 (0.04) | -6.0 (0.1) |

^a^Errors for displacement experiments are average deviations of 2-5 measurements. Errors for direct titrations are standard deviations from the non-linear least squares curve fits.
