# Supplementary material for: Testing Electrostatic Complementarity in Enzyme Catalysis: Hydrogen Bonding in the Ketosteroid Isomerase Oxyanion Hole
Source: PLoS Biol. 2006 Mar 28;4(4):e99. doi: 10.1371/journal.pbio.0040099 (PMC1413570; doi:10.1371/journal.pbio.0040099)
Supplement: Supplementary file 13 [file pbio.0040099.st004.doc]

**Table S4**. p*K*_a_ values for phenols used in this work^a^.

| **Phenol** | **p*K*_a_** | **Phenol** | **p*K*_a_** |
| --- | --- | --- | --- |
| 3,4-(NO_2_)_2_ | 5.4 | 3-CF_3_ | 8.7^b^ |
| 3-F-4-NO_2_ | 6.1^b^ | 2-F | 8.8 |
| 3-CF_3_-4-NO_2_ | 6.3^b^ | 4-CF_3_ | 8.9^b^ |
| 4-NO_2_ | 7.1 | 3-Cl | 9.0 |
| 2,3,5-F_3_ | 7.1^b^ | 3,4-F_2_ | 9.1^b^ |
| 2,3,4-F_3_ | 7.7^b^ | 3-Br | 9.1 |
| 2,4,5-F_3_ | 7.7^b^ | 3-I | 9.2 |
| 2,5-F_2_ | 7.7^b^ | 3-F | 9.3 |
| 2,3-F_2_ | 7.7^b^ | 4-I | 9.3 |
| 3-NO_2_-4-Cl | 7.8^b^ | 4-Br | 9.3 |
| 4-CN | 8.0 | 4-Cl | 9.4 |
| 3,5-Cl_2_ | 8.1^b^ | 4-F-3-CH_3_ | 9.8^b^ |
| 4-acetyl | 8.1 | 3-ethyl | 9.9 |
| 3,4,5-F_3_ | 8.2^b^ | 3-isopropyl | 9.9^b^ |
| 3-F-5-CF_3_ | 8.2^b^ | 4-F | 10.0 |
| 3,5-F_2_ | 8.4^b^ | Phenol | 10.0 |
| 3-NO_2_ | 8.4 | 4-ethyl | 10.0 |
| 3,4-Cl_2_ | 8.5^b^ | 4-isoproyl | 10.0^b^ |
| 2,4-F_2_ | 8.6^b^ | 3-methyl | 10.1 |
| 3-CN | 8.6 | 4-methyl | 10.2 |

^a^From [137] unless otherwise noted.

^b^Determined by spectral titration.
