# Supplementary material for: Testing Electrostatic Complementarity in Enzyme Catalysis: Hydrogen Bonding in the Ketosteroid Isomerase Oxyanion Hole
Source: PLoS Biol. 2006 Mar 28;4(4):e99. doi: 10.1371/journal.pbio.0040099 (PMC1413570; doi:10.1371/journal.pbio.0040099)
Supplement: Supplementary file 14 [file pbio.0040099.st005.doc]

**Table S5**. Heats of ionization of phenols.

| **Phenol** | **p*K*_a_** | **Ionic strength** | **∆H_ion_** | **Lit value**^a^ |
| --- | --- | --- | --- | --- |
| 4-F | 10.0 | 0.0 | 5.97 (0.03) | 5.93 (0.05) |
| 4-F | 10.0 | 0.1 | 6.21 (0.06) |  |
| Phenol | 10.0 | 0.1 | 6.55 (0.05) | 6.1 (0.2) |
| 3-F | 9.3 | 0.1 | 6.29 (0.05) |  |
| 3,4-F_2_ | 9.1 | 0.1 | 7.04 (0.06) |  |
| 3,5-F_2_ | 8.4 | 0.1 | 5.73 (0.07) |  |
| 3,4,5-F_3_ | 8.2 | 0.1 | 5.62 (0.06) |  |

^a^From [148].
